# A Robust Heterometallic Pt_2_Pd_2_L_8_ Double Cage Catenane

**DOI:** 10.1002/anie.202516952

**Published:** 2025-09-30

**Authors:** Sudhakar Ganta, Alexander S. Mikherdov, Ananya Baksi, Christoph Drechsler, Guido H. Clever

**Affiliations:** ^1^ Department of Chemistry and Chemical Biology TU Dortmund University Otto‐Hahn Straße 6 44227 Dortmund Germany; ^2^ Department of Chemistry Jadavpur University Raja S.C. Mallick Rd. 188 Kolkata 700032 India

**Keywords:** Coordination cages, Halide abstraction, Heterometallic structures, Mechanically interlocked molecules, Supramolecular chemistry

## Abstract

The combination of different metal ions in supramolecular structures can lead to the emergence of new features, such as enhanced stability, reduced symmetry, and peculiar reactivity. Mechanically interlocked architectures containing dissimilar metal ions are still scarce. Herein, we report the first example of a heterometallic, quadruply interlocked Pt_2_Pd_2_ **L**_8_ cage catenane, assembled via a combination of metal‐mediated self‐assembly and dynamic covalent chemistry. While direct complexation of the used asymmetric ligand with a mixture of Pt(II) and Pd(II) ions could yield 14 different monomeric and 576 interlocked cage products, the chosen approach allowed us to selectively obtain one PtPd**L**_4_ monomeric cage and a single Pt_2_Pd_2_ **L**_8_ double cage isomer as major products, respectively, depending on the reaction conditions. In the obtained interlocked Pt_2_Pd_2_ **L**_8_ structure, the kinetically inert Pt(II) ions are positioned peripherally, while the more labile Pd(II) ions are buried and interlocked within the cage interior, as confirmed by NMR spectroscopy, trapped ion mobility spectrometry (TIMS), and single‐crystal X‐ray diffraction. The resulting catenane structure exhibits an enhanced kinetic stability, resisting disassembly in the presence of excess competitive ligands such as halide anions, unlike previously reported Pd(II) cage catenanes. Furthermore, the Pt_2_Pd_2_ **L**_8_ cage binds halide anions with high affinity, enabling efficient sequestration of halides from organic substrates.

## Graphical abstract

The first heterometallic Pt(II)–Pd(II) quadruply interlocked cage catenane (Pt_2_Pd_2_L_8_) has been assembled via a combination of metal‐mediated self‐assembly and dynamic covalent chemistry. The integration of inert Pt(II) and labile Pd(II) ions within the architecture enables facile catenation while strongly enhancing stability of the assembly.

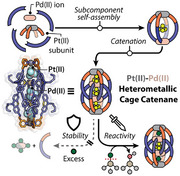

Metal‐templated and metal‐mediated self‐assembly offer efficient ways for generating mechanically interlocked structures such as catenanes, rotaxanes, and knots with diverse topologies and varying degrees of interlocking.^[^ ^1^, ^2^, ^3^, ^4^, ^5^, ^6^, ^7^ ^]^ Pioneering work by Fujita has demonstrated that the self‐assembly of ten components can quantitatively result in triply interlocked cage catenanes based on either Pd(II) or Pt(II) nodes, with the latter metal strongly enhancing the product's stability.^[^ ^8^ ^]^ In the case of Pd(II)‐mediated self‐assemblies, it has been repeatedly observed by Kuroda, Hiraoka, our group, and others that lantern‐shaped Pd_2_L_4_ coordination cages can dimerize to form *D*_4_‐symmetric Pd_4_L_8_ double cages with an even higher degree of topological entangling, i.e., quadruply interlocked cage catenanes (Figure 1a).^[^ ^2^, ^5^, ^7^ ^]^ The formation of mechanical bonds in these systems not only leads to non‐trivial supramolecular architectures but also imparts them with new capabilities, such as selective guest recognition,^[^ ^9^, ^10^ ^]^ allosteric binding,^[^ ^11^ ^]^ anion‐based catalytic activity,^[^ ^12^ ^]^ photoinduced charge separation,^[^ ^13^ ^]^ photosensitization of small molecules,^[^ ^14^ ^]^ stimuli‐responsive behavior,^[^ ^15^ ^]^ and switchability in multistate networks.^[^ ^16^ ^]^

**Figure 1:**
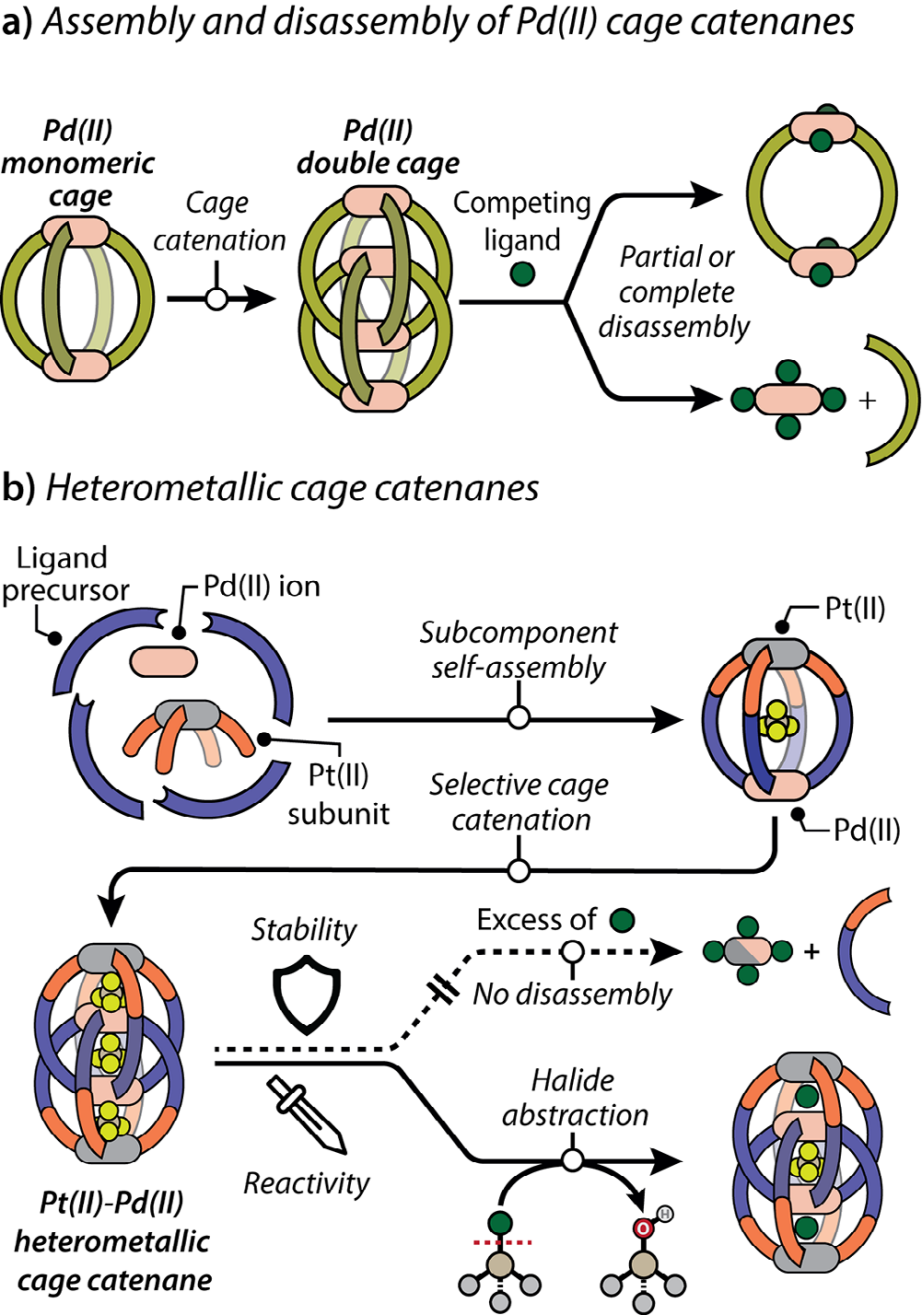
Self‐assembly, stability, and reactivity of quadruply interlocked a) purely Pd(II) and b) heterometallic Pt(II)‐Pd(II) cage catenanes.

The ease of formation of Pd(II)‐based interlocked structures originates from the relatively labile and dynamic nature of the Pd(II)–L (usually L = pyridine) coordination bonds, which allows initially formed simpler and smaller Pd(II) assemblies to reconstruct into more complex interlocked species once certain thermodynamic key criteria are fulfilled.^[^ ^2^, ^5^, ^6^ ^]^ However, the same lability comes at the cost of reduced kinetic and thermodynamic stability of the final assemblies. For instance, quadruply interlocked Pd_4_L_8_ cage catenanes can undergo partial or complete disassembly in the presence of excess competing ligands or Lewis or Brønsted acids (Figure 1a). ^[^ ^11^, ^12^, ^16^, ^17^, ^18^ ^]^ The utilization of chemically similar (*square‐planar* coordination and comparable ionic radius) but more kinetically inert Pt(II) ions can indeed drastically enhance the stability of a metallo‐supramolecular system but often hampers the clean and reasonably fast assembly of interlocked topologies that form through iterative bond formation and breaking processes.^[^ ^19^, ^20^, ^21^ ^]^ To date, only singly interlocked Pt(II)‐based ring catenanes^[^ ^19^, ^20^ ^]^ and a few singly‐ or triply‐interlocked Pt(II) cage catenanes^[^ ^8^, ^22^, ^23^ ^]^ have been reported in the literature, with no examples of more highly intertwined structures.

Combining different metal ions in a supramolecular structure can be advantageous to unite the benefits of a high‐yielding, dynamic assembly process (promoted by the more kinetically accessible metal ion) with an enhanced overall stability of the final product (as a function of the more inert metal component). Furthermore, it can serve to reduce the assembly's symmetry^[^ ^24^ ^]^ and give rise to new properties, such as catalytic activity.^[^ ^25^ ^]^ Several approaches for obtaining non‐entangled heterometallic cages containing *square‐planar* metal ions have been reported, such as subcomponent self‐assembly (e.g., Pt(II)‐Fe(II) and Pt(II)‐Zn(II) cages by Nitschke^[^ ^26^ ^]^ and Lützen,^[^ ^27^ ^]^ Pt(II)‐Pd(II)^[^ ^28^ ^]^ and Ru(II)‐Pd(II)^[^ ^29^ ^]^ cages by Crowley), metal–donor site matching using unsymmetric ligands (e.g., Pt(II)‐Pd(II) cages by Crowley^[^ ^30^ ^]^ and Cu(II)‐Pd(II) cages by Tuck and Turner^[^ ^31^ ^]^), and post‐assembly modification (e.g., Cu(II)‐Pd(II) cages by Bloch^[^ ^32^ ^]^). The construction of heterometallic mechanically interlocked structures is even more challenging. Previously, Mukherjee and coworkers attempted to obtain heterometallic triply interlocked cage catenanes by mixing Pd(II) and Pt(II) homometallic monomeric cages.^[^ ^22^ ^]^ However, the system underwent self‐sorting, resulting in homometallic interlocked cage structures. To the best of our knowledge, no cage catenanes with heterometallic nodes have been reported to date.

In this work, we report the synthesis of the first heterobimetallic Pt(II)‐Pd(II) quadruply interlocked cage catenane (Pt_2_Pd_2_ **L**_8_) by combining the subcomponent self‐assembly of a pre‐formed Pt(II) fragment with ligand counterparts fulfilling previously elaborated design principles (length, donor angle, steric demand) to allow for quadruply interlocked cage formation in a proper solvent and counter anion environment (Figure 1b). The incorporation of both labile Pd(II) and inert Pt(II) ions within the same cage structure still allows facile cage catenation while also enhancing the overall stability of the final assembly compared to analogous purely Pd(II)‐based catenated species. We demonstrate the enhanced chemical stability of the mixed‐metal cage toward excess halide anions, as compared to structures only containing Pd(II) that usually fall apart when halide substitution of pyridyl ligands at the metal centers outcompetes overall supramolecular assembly stability. On the other hand, we show that the Pt_2_Pd_2_ **L**_8_ interpenetrated dimer still shows a high affinity for allosterically binding two halide anions in its outer pockets, similar to its Pd(II)‐based progenitors, and demonstrate its applicability for the effective abstraction of halides from haloorganics, converting them into the corresponding alcohols in the presence of water.

To exclusively obtain a defined heterometallic cage, the ligand design should imply the presence of two distinct donor groups, able to selectively coordinate to Pd(II) and Pt(II) ions, respectively.^[^ ^24^ ^]^ This is, however, not easily achievable, given the essentially identical coordination geometry and donor preferences of these two closely related cations. The herein designed asymmetric ligand **L** (prepared by the imine condensation of 10‐hexyl‐7‐(pyridin‐3‐ylethynyl)‐10H‐phenothiazin‐3‐amine (**PTA**) with 3‐pyridylcarbaldehyde (**PyCHO**); Figure 1a) shares structural similarities to many previously employed banana‐shaped ligands^[^ ^33^ ^]^ with the difference that the tricyclic backbone connects to the pyridine donors via one ethinyl and one imine linker, slightly deviating in length (4.0 Å versus 3.7 Å) and geometry. The direct complexation of **L** with only Pd(II) ions can result in a mixture of four possible isomeric Pd_2_ **L**_4_ cages (with different **L** orientations, Figure 2a),^[^ ^34^ ^]^ since the only slight ligand asymmetry with identical donors on both sides is not sufficient to promote self‐sorting to a distinct isomer as compared to the systems described by Yuasa,^[^ ^35^ ^]^ Lewis,^[^ ^36^ ^]^ Crowley,^[^ ^37^ ^]^ or Chand.^[^ ^38^ ^]^ Further catenation of these four cages could in principle yield a mixture containing up to 42 isomeric Pd_4_ **L**_8_ double cages (including enantiomers and *hetero*‐interlocked structures—formed from different monomeric cage isomers;^[^ ^13^ ^]^ for details, see Scheme S2). The formation of a complex mixture was then indeed supported by NMR spectroscopy and trapped ion mobility spectrometry (TIMS), while the analytical data was too convoluted to allow for differentiating or quantifying individual species (see Section S2.2 for details). For comparison, a pseudo‐tetrahedral cage Pd_4_ **L**_8_ of the same stoichiometry based on an asymmetric ligand reported by Severin et al. was calculated to give 35 possible isomers.^[^ ^39^ ^]^ Similarly, the direct reaction of **L** with a mixture of Pd(II) and Pt(II) ions would also not be expected to proceed under any geometrical control^[^ ^40^ ^]^ and would result in an even greater variety of products: four homometallic Pd_2_ **L**_4_ and four homometallic Pt_2_ **L**_4_ cages, along with six heterometallic PtPd**L**_4_ cages. Upon catenation, these 14 cages could theoretically form 105 possible pairs (14 for *homo*‐interlocked and 91 for *hetero*‐interlocked), leading to 576 potential quadruply interlocked cage products: 42 Pd_4_ **L**_8_, 42 Pt_4_ **L**_8_, 136 PtPd_3_ **L**_8_, 136 Pt_3_Pd**L**_8_, and 220 Pt_2_Pd_2_ **L**_8_ structures (Schemes S2–S5).

**Figure 2:**
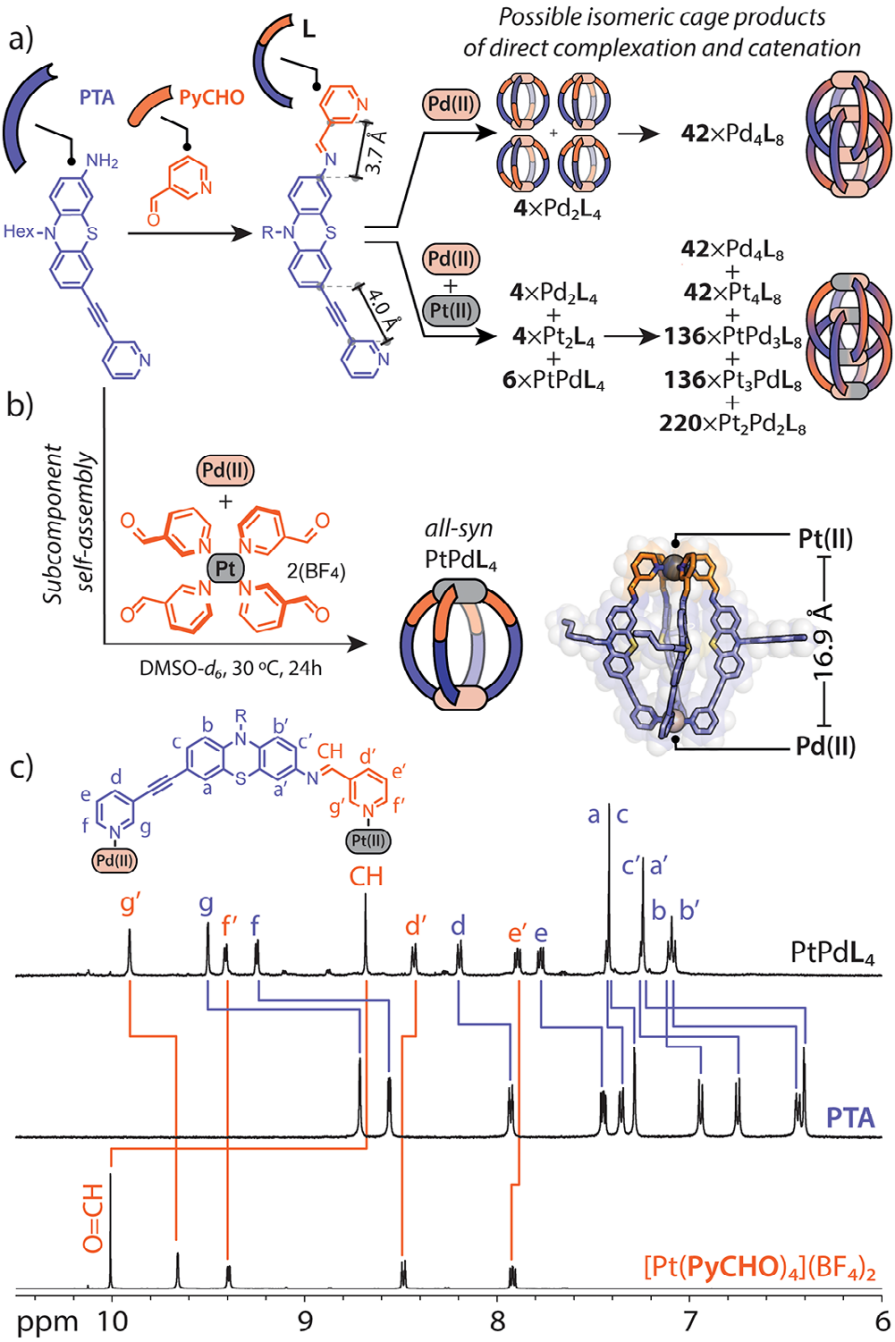
a) Formation of asymmetric ligand **L** from **PTA** and **PyCHO** and theoretical outcome of its uncontrolled assembly with Pd(II) and Pt(II) ions (note that most interlocked Pt(II)‐rich species would be kinetically difficult to access). b) Self‐assembly of a single isomer of cage PtPd**L**_4_ from **PTA**, [Pt(**PyCHO**)_4_](BF_4_)_2_, and [Pd(CH_3_CN)_4_](BF_4_)_2_ in DMSO‐*d_6_* and its DFT model. c) Partial ^1^H NMR (500 MHz, DMSO‐*d_6_*) spectra of PtPd**L**_4_ (top), **PTA** (middle), [Pt(**PyCHO**)_4_](BF_4_)_2_ (bottom).

We thus had to devise a strategy aimed at precisely placing the Pd(II) and Pt(II) cations and achieved this through combining Pd(II)‐mediated self‐assembly with dynamic covalent imine condensation involving a pre‐formed Pt(II) subunit (subcomponent self‐assembly approach)^[^ ^28^, ^41^ ^]^ under conditions that promote cage dimerization. First, the one‐pot reaction of ligand precursor **PTA** (4 eq.) with pre‐formed complex [Pt(**PyCHO**)_4_](BF_4_)_2_ (1.1 eq.), in the presence of palladium source [Pd(CH_3_CN)_4_](BF_4_)_2_ (1 eq.), was studied in DMSO‐*d_6_* and yielded after 24 h at 30 °C exclusively the all‐*syn* heterometallic monomeric PtPd**L**_4_ cage isomer (Figure 2b), where the Pd(II) ion is bound by the ethynyl‐connected pyridines, while the Pt(II) ion is coordinated to those connected by the imine functionalities. The ^1^H NMR spectrum of the cage shows only one set of signals corresponding to incorporated ligand **L** with a general downfield shift of aromatic signals compared to those of the free subcomponents (Figure 2c). Notably, the signals at 10.00 and 4.91 ppm, corresponding to the aldehyde and amine protons of the initial components, disappeared, while a new characteristic signal for the imine proton emerged at 8.68 ppm, providing clear evidence for the formation of imine bonds in the heterometallic PtPd**L**_4_ assembly. ^1^H DOSY NMR confirmed the presence of a single species with a calculated hydrodynamic radius (*r*_H_) of 12.8 Å (Figure S15), while high‐resolution ESI‐MS further confirmed the PtPd**L**_4_ stoichiometry of the cage, revealing a characteristic isotope pattern corresponding to the major [PtPd**L**_4_]^4+^ ion (*m/z* 563.4, Figure S16).^[^ ^33^ ^]^

Next, the self‐assembly of **PTA** with [Pt(**PyCHO**)_4_](BF_4_)_2_ and a Pd(II) source in the same ratio as described above was investigated in CD_3_CN as a solvent to promote the dimerization and in situ interlocking of the monomeric cages (Figure 3a).^[^ ^2^ ^]^ Indeed, after reacting the mixture for 48 h at 30 °C, the ^1^H NMR spectrum showed a single major species with only one set of signals assignable to the protons of ligand **L** (Figure 3b). In the ^1^H DOSY NMR spectrum, all these signals belong to a single diffusion coefficient, corresponding to an *r*_H_ value of 14.7 Å, which is larger than that of the monomeric PtPd**L**_4_ cage and supports the formation of a double cage structure (Figure S20).^[^ ^11^, ^17^ ^]^ Using the signal of non‐deuterated acetonitrile (originating from precursor [Pd(CH_3_CN)_4_](BF_4_)_2_) as an internal standard, the fraction of the major species was estimated to be about 70%. The ESI‐MS analysis further confirmed the formation of a heterometallic Pt_2_Pd_2_ **L**_8_ double cage, as evidenced by the detection of signals corresponding to the [Pt_2_Pd_2_ **L**_8_ + *n*BF_4_]^(8–^ *^n^* ^)+^ (*n* = 3, 4, 5) ions with *m/z* 954.3, 1214.6, and 1648.5, respectively (Figure 3c). Interestingly, online monitoring of the reaction between the subcomponents in CD_3_CN by ^1^H NMR revealed that during the cage assembly at first the disappearance of **PTA** signals and emergence of new signals corresponding to monometallic Pd*_n_* (**PTA**)_2_ *_n_* (*n* = 2, 3) assemblies (which were also obtained and characterized separately; see Section S3.1 for details) were observed. Then, on a somewhat slower timescale, the resulting Pd(II) assemblies react with the [Pt(**PyCHO**)_4_](BF_4_)_2_ building block under imine condensation, ultimately leading to the sole formation of heterobimetallic species.^[^ ^42^ ^]^

**Figure 3:**
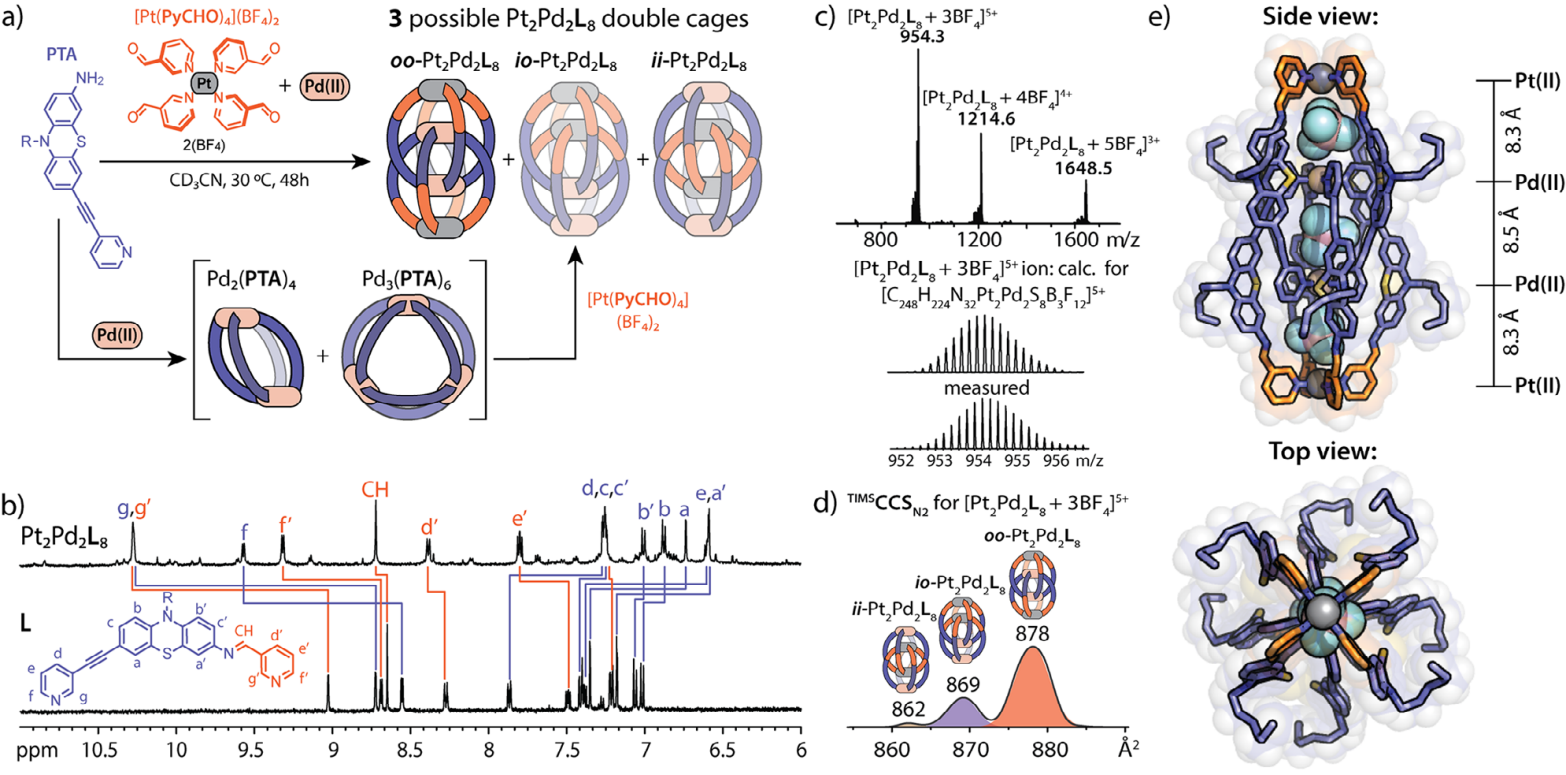
a) Self‐assembly of the major *oo*‐Pt_2_Pd_2_ **L**_8_ double cage isomer from **PTA**, [Pt(**PyCHO**)_4_](BF_4_)_2_, and [Pd(CH_3_CN)_4_](BF_4_)_2_ in CD_3_CN; b) Partial ^1^H NMR (500 MHz, CD_3_CN) spectra of Pt_2_Pd_2_ **L**_8_ (top) and **L** (bottom); c) ESI‐MS of heterometallic Pt_2_Pd_2_ **L**_8_ (top), calculated and measured isotopic distribution for the [Pt_2_Pd_2_ **L**_8 _+ 3BF_4_]^5+^ ion (bottom); d) TIMS mobilogram for the [Pt_2_Pd_2_ **L**_8 _+ 3BF_4_]^5+^ ion (experimental CCS values for three observed isomers given); e) Side and top views of the X‐ray structure of quadruply interlocked *oo*‐Pt_2_Pd_2_ **L**_8_ double cage with inter‐metal distances.

Due to the asymmetric structure of the monomeric PtPd**L**_4_ cage, its dimerization in CD_3_CN can possibly result in three supramolecular isomers of quadruply interlocked heterometallic Pt_2_Pd_2_ **L**_8_ double cages based on the positions of the Pt(II) and Pd(II) ions within the cage: a symmetric *oo*‐Pt_2_Pd_2_ **L**_8_ cage with Pt atoms outside (*o*) the cage interior, a symmetric *ii*‐Pt_2_Pd_2_ **L**_8_ structure with Pt atoms inside (*i*), and an asymmetric *io*‐Pt_2_Pd_2_ **L**_8_ isomer with one Pt and one Pd atom each inside and outside (Figure 3a). A deeper analysis of the NMR and MS data allowed us to determine the structure of the major self‐assembly product in CD_3_CN. First, the observation of a single set of ligand signals in the ^1^H NMR spectrum can only correspond to a *D*_4_‐symmetric double cage structure such as *oo*‐ or *ii*‐Pt_2_Pd_2_ **L**_8_, since the asymmetric *io*‐Pt_2_Pd_2_ **L**_8_ structure (only *C*_4_ symmetry) would exhibit a twofold splitting of ligand signals. Second, in comparison to free ligand **L**, the *ortho*‐proton signals of the Py moieties coordinated to the Pd(II) atoms showed a generally larger downfield shift (Δ*δ* ca. 1.5 and 1.0 ppm for ***g*** and ***f*** protons) than those of the Py groups coordinated to Pt(II) (Δ*δ* ca. 1.2 and 0.6 ppm for ***g'*** and ***f'*** protons; Figure 3b). At the same time, the signal of the protons from the imine groups located near the Pt(II) ions remains almost unaltered (Δ*δ* − 0.07 ppm) compared to free ligand **L**. Based on previous reports on comparable Pd_4_L_8_ double cages, this suggests that the Pd atoms and coordinated Py groups, rather than the Pt environments, are located inside the assembly.^[^ ^9^, ^11^, ^17^, ^18^, ^43^ ^]^ Additionally, ^1^H‐^1^H NOESY measurements reveal a NOE interaction between ***g*** and ***a*** protons of ligand **L**, which is most consistent with the *oo*‐Pt_2_Pd_2_ **L**_8_ structure (Figure S19). Finally, in addition to the ESI‐MS measurements, we analyzed the major [Pt_2_Pd_2_ **L**_8_ + 3BF_4_]^5+^ ion using TIMS, which can discriminate between isomeric cage structures based on their size and shape via their collisional cross‐sections (CCS; Figure 3d).^[^ ^44^, ^45^, ^46^ ^]^ TIMS analysis revealed three distinct mobility signals with CCS values of 878, 869, and 862 Å^2^, indicating the presence of all three possible isomeric Pt_2_Pd_2_ **L**_8_ double cages. The theoretically calculated ^theo^CCS_N2_ values for the modelled structures of *oo*‐ (984 Å^2^), *io*‐ (936 Å^2^), and *ii*‐ (922 Å^2^) Pt_2_Pd_2_ **L**_8_ are – while somewhat overshooting—consistent with the trend observed in the experimental TIMS results. Based on these data, the isomer ratio in the gas phase can be determined to be approximately 75:22:3 (*oo*:*io*:*ii*), suggesting the *oo*‐isomer (^TIMS^CCS_N2_ = 878 Å^2^) as the major component, consistent with the NMR results.

**Figure 4:**
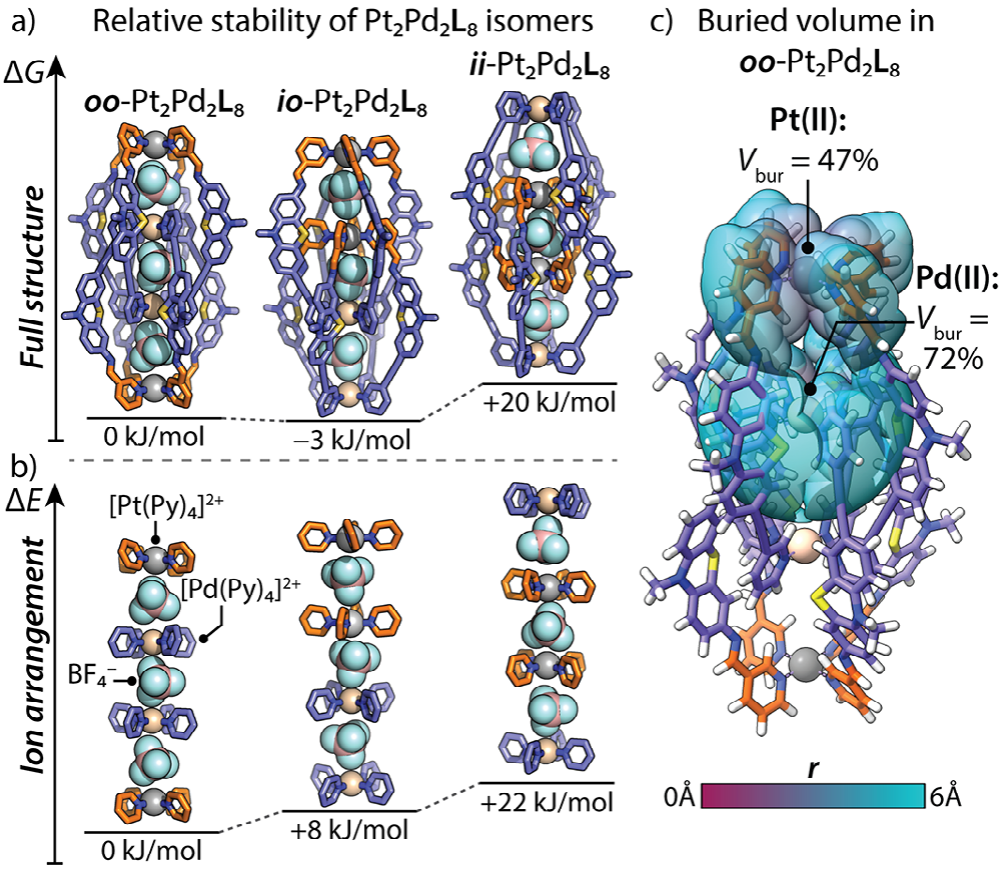
Optimized structures of *oo*‐, *io*‐, *ii*‐Pt_2_Pd_2_ **L**_8_ isomers, their fragments, and relative energies of the a) full structures and b) fragments. Data is taken from Table S3. c) Visualization of buried volume (*V*_bur_) for optimized *oo*‐Pt_2_Pd_2_ **L**_8_ structure around Pt(II) and Pd(II) metal centers in the radius of 6 Å from them. Visualization and buried volume analysis are performed using UCSF ChimeraX 1.9 software^[^ ^55^ ^]^ with the SEQCROW bundle.^[^ ^56^ ^].^

We also managed to grow single crystals of the Pt_2_Pd_2_ **L**_8_ assembly suitable for X‐ray diffraction analysis using vapor diffusion of benzene into a CD_3_CN cage solution in the presence of *n*‐Bu_4_NCl as an additive. The obtained solid‐state structure indeed corresponds to the quadruply interlocked *oo*‐Pt_2_Pd_2_ **L**_8_ isomer, which agrees with the structure proposed from the solution and gas‐phase studies (Figure 3e). Three BF_4_^–^ counter‐ions were found encapsulated in the cage cavities: two in the outer and one in the inner pocket.^[^ ^17^ ^]^ The observed inter‐metal distances across the cavities (8.3–8.5 Å) also agree with BF_4_^–^ encapsulation^[^ ^9^, ^16^ ^]^ and are only slightly longer than in the optimized structure (8.1–8.4 Å). To our surprise, one Cl^–^ counter‐anion (coming from the *n*‐Bu_4_NCl additive) was found outside the cage despite the exceptionally high affinity of these double cages to encapsulate halide anions.^[^ ^11^ ^]^ This single chloride anion was, however, found to be tightly bound between peripheral Pt(Py)_4_ units of two neighboring double cages and probably acts as a crucial building block in the solid‐state assembly, reinforcing the crystal lattice by inter‐cage ion‐pairing interactions (Figure S32). The corresponding Pt‐Pt separation of 7.1 Å is also appropriate for chloride encapsulation in the pocket formed between two cages in the solid‐state structure.^[^ ^9^, ^16^ ^]^

To gain further insight into the supramolecular isomerism of the heterometallic Pt_2_Pd_2_ **L**_8_ double cages, we prepared theoretical models of the *oo*‐, *io*‐, and *ii*‐isomeric structures with three encapsulated BF_4_^−^ anions (Figure 4a; geometry optimization at the r^2^scan‐3c^[^ ^47^ ^]^/SMD^[^ ^48^ ^]^(CH_3_CN) level) and theoretically investigated their relative stabilities (single‐point energy calculations at the ωB97X^[^ ^49^ ^]^‐D4^[^ ^50^ ^]^/def2TZVPP^[^ ^51^ ^]^/SMD^[^ ^48^ ^]^‐DRACO^[^ ^52^ ^]^(CH_3_CN) level with thermostatistical corrections by the mRRHO approach^[^ ^53^ ^]^; see Section S7 and Table S3). According to the results, the *oo*‐Pt_2_Pd_2_ **L**_8_ isomer (observed in the solution and solid state) is approximately 20 kJ mol^−1^ more stable than the other symmetric *ii*‐isomer and only ca. 3 kJ mol^−1^ less stable than the asymmetric *io*‐isomer. Fragmentation analysis of the structures and comparison of fragment energies across the isomers revealed that the linear alternating arrangement of four [M(Py)_4_]^2+^ and three BF_4_^−^ ions (excluding the ligand backbones, Figures 4b and S34) with distances fixed as in the full models is 8 and 22 kJ mol^−1^ more favorable in the *oo*‐isomer than in the *io*‐ and *ii*‐isomers, respectively, which probably adds the major contribution to the *oo*‐isomer stabilization. Conversely, the ligand arrangement (excluding the metals and BF4^−^ moieties) is more favorable in the *io*‐ and *ii*‐isomers by 20 and 8 kJ mol^−1^ than in the *oo*‐isomer (Figure S33 and Table S3). Although the calculations suggest that the *io*‐isomer may be thermodynamically slightly more stable than the experimentally confirmed *oo*‐isomer, the formation of the *io*‐isomer is expected to be kinetically more hindered because the multiple ligand de‐ and recoordination events required for the interlocking of two monomeric cages to give the dimer are less probable when a Pt(II) ion is located inside the cage interior (see Section S3.3 for discussion).^[^ ^54^ ^]^

Due to its peculiar structural features, the observed *oo*‐Pt_2_Pd_2_ **L**_8_ isomer was expected to exhibit greater kinetic stability toward the addition of competitive ligands than previously reported purely Pd(II)‐based monomeric and interlocked double cages. In particular, enhanced stability should arise from the placement of the more kinetically inert Pt(II) at the peripheral positions, while the more labile Pd(II) ions become buried inside the cage interior (Figure 4c), sterically hindering attack by competing ligands (followed by ligand displacement following an associative exchange mechanism). To test this hypothesis, we investigated the behavior of the Pt_2_Pd_2_ **L**_8_ cage in the presence of halide anions (Cl^–^, Br^–^) as competitive ligands (Figure 5a).

**Figure 5:**
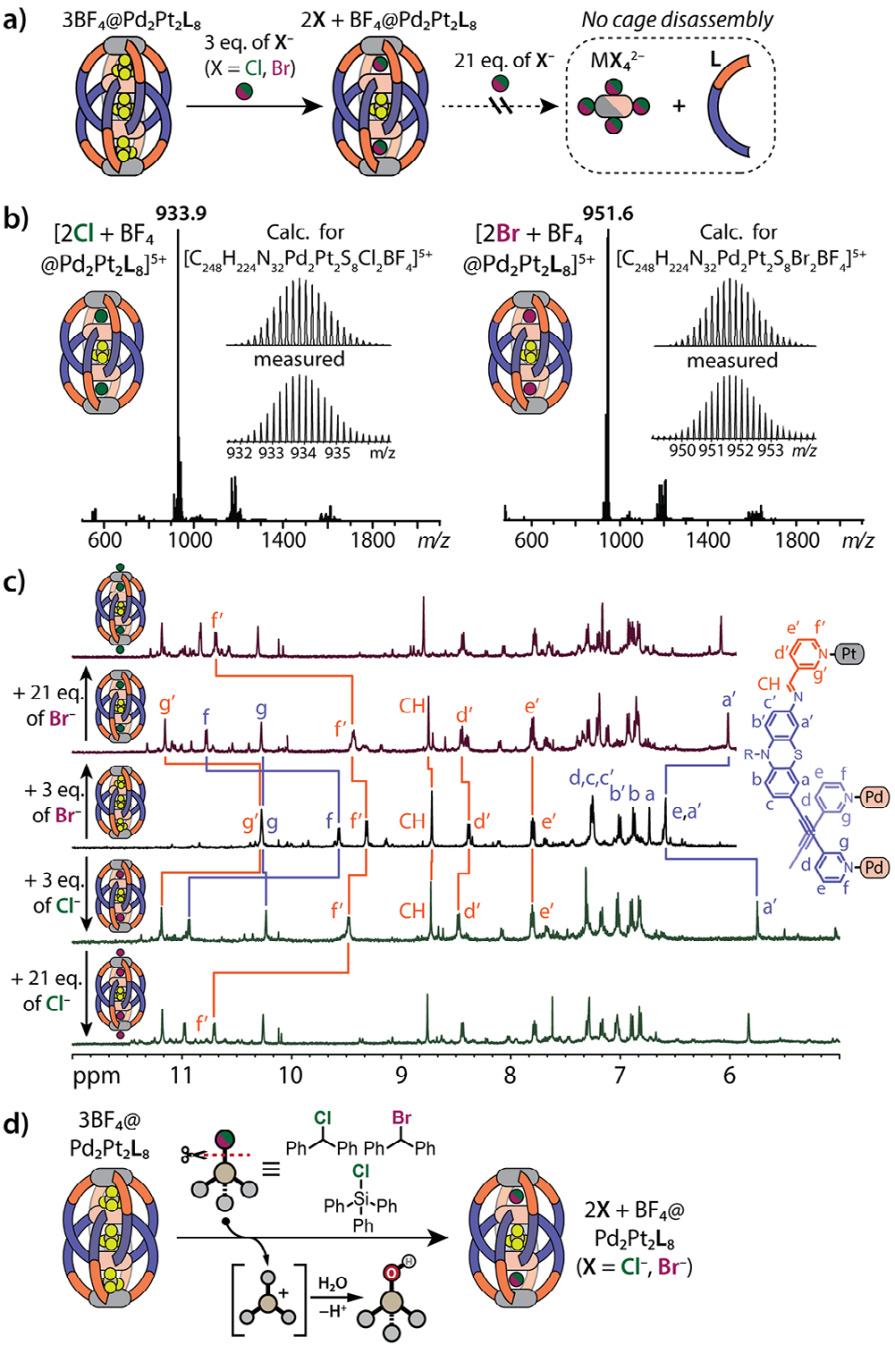
a) Interaction of the Pt_2_Pd_2_ **L**_8_ cage with halide anions. b) ESI‐MS of 2Cl + BF_4_@Pt_2_Pd_2_ **L**_8_ (left) and 2Br + BF_4_@Pt_2_Pd_2_ **L**_8_ (right). c) ^1^H NMR (500 MHz, CD_3_CN, 298 K) spectra of 3BF_4_@Pt_2_Pd_2_ **L**_8_ (middle spectrum) double cages, after addition of 3 and 24 eq. of *n*‐Bu_4_NCl (bottom spectra), and after addition of 3 and 24 eq. of *n*‐Bu_4_NBr (top spectra). d) Halide abstraction by Pt_2_Pd_2_ **L**_8_ double cage from organic molecules.

Reported similar interlocked Pd_4_L_8_ double cages are known for their exceptionally high binding affinity toward halide anions. However, the addition of excess halides (more than 3 eq.) as competitive ligands often leads to partial or complete double cage disassembly, thus hampering their applicability as halide abstracting reagents or sensors.^[^ ^9^, ^11^, ^12^, ^18^ ^]^ The titration of Pt_2_Pd_2_ **L**_8_ with 1–3 eq. of chloride and bromide anions (as their *n*‐Bu_4_N^+^ salts) initially results in the encapsulation of two halide anions, replacing the BF_4_^–^ anions in the outer cavities (Figure 5a). The transformation of the 3BF_4_@Pt_2_Pd_2_ **L**_8_ assembly into the 2X + BF_4_@Pt_2_Pd_2_ **L**_8_ species was first confirmed by ESI‐MS analysis (Figure 5b). Interestingly, TIMS measurements of the [2X + BF_4_@Pt_2_Pd_2_ **L**_8_]^5+^ ions for both assemblies still revealed the presence of three species with distinct CCS values, suggesting the coexistence of the major with the two minor isomers, all able to encapsulate two halide anions (Figure S29). The CCS values obtained for the [2X + BF_4_@Pt_2_Pd_2_ **L**_8_]^5+^ ions are smaller than those for the [3BF_4_@Pt_2_Pd_2_ **L**_8_]^5+^ ions, indicating a structural contraction of the double cage upon binding two halide guests, in full accordance with our previous studies.^[^ ^9^, ^11^ ^]^

In the ^1^H NMR spectra, the addition of up to 3 eq. of halide anions causes a significant downfield shift of the pyridine ***g'*** and ***f*** protons, which point inward to the outer cavities of the *oo*‐Pt_2_Pd_2_ **L**_8_ cage (Figure 5c). Additionally, the inward‐pointing phenothiazine ***a*** and ***a'*** protons exhibit noticeable upfield and downfield shifts, respectively. At the same time, the ***g*** protons of the pyridine moieties connected to the Pd(II) ions located close to the inner cavity remained mostly unaffected. Consistent with ESI‐MS results, the NMR data thus also suggest that only the two BF_4_^–^ anions in the outer cavities of *oo*‐Pt_2_Pd_2_ **L**_8_ undergo exchange with halide anions.

As anticipated, the further addition of halide anions (up to 24 eq.) did not lead to any signs of disassembly of the Pt_2_Pd_2_ **L**_8_ double cage, indicating its higher stability compared to monometallic Pd(II)‐based monomeric and double cages. The most notable change in the ^1^H NMR spectra upon excess addition of halides is observed for the outward‐pointing ***f'*** proton of the pyridine groups coordinated to the peripheral Pt(II) ions. This signal is gradually shifting downfield by more than 1 ppm, suggesting *exo*‐binding of halide anions to the peripheral Pt(Py)_4_ units of the *oo*‐Pt_2_Pd_2_ **L**_8_ cage.^[^ ^57^ ^]^ While the addition of halides did not result in any noticeable decrease in the *oo*‐isomer signal intensity, the signals of other minor species were observed to shift and split. At 24 eq. of either chloride or bromide added, small signals appear with shifts similar (but not identical) to the free ligand that may be assigned to Pd‐decomplexed fragments of the cage, suggesting a lower stability of the minor Pt_2_Pd_2_ **L**_8_ isomers. While these isomers, featuring Pd complexes on the periphery, should be kinetically more prone to halide‐triggered disassembly, DFT calculations (Table S5) further indicate that the *oo*‐isomer with two encapsulated halides may also be thermodynamically more stable (by more than 30 kJ mol^−1^) than the *io*‐ and *ii*‐isomers containing halides in their outer pockets, further supporting the hypothesis that the minor isomers are more prone to disassembly in the presence of competing ligands than the *oo*‐isomer.

The combination of kinetic stability and strong affinity of the heterometallic *oo*‐Pt_2_Pd_2_ **L**_8_ cage toward halide anions makes it a promising candidate for capturing halides from various halogenated compounds via halide abstraction reactions.^[^ ^12^, ^58^, ^59^ ^]^ To demonstrate this, we studied the behavior of the Pt_2_Pd_2_ **L**_8_ cage in the presence of benzhydryl halides and chlorotriphenylsilane (Figure 5d). The reaction of the cage with 2 eq. of benzhydryl chloride at 70 °C led to halide abstraction from it and formation of the 2Cl + BF_4_@Pt_2_Pd_2_ **L**_8_ species, as evidenced by ^1^H NMR (Figure S30). Additionally, ESI‐MS analysis indicated the presence of the benzhydryl cation (Ph_2_CH^+^, *m/z* = 167.1; Figure S31) besides signals corresponding to the [2Cl + BF_4_@Pt_2_Pd_2_ **L**_8 _+ *n*BF_4_]^(5–^ *^n^* ^)+^ ions. This then further reacts to benzhydrol in the presence of residual water from the solvent.^[^ ^12^, ^58^, ^59^ ^]^ At the same time, the utilization of more reactive benzhydryl bromide and chlorotriphenylsilane resulted in similar halide abstraction reactions and hydrolysis in the presence of the Pt_2_Pd_2_ **L**_8_ cage already at room temperature (Figure S30).

In summary, we report the first example of a heterometallic quadruply interlocked Pt_2_Pd_2_ **L**_8_ cage catenane, obtained through a combination of metal‐mediated self‐assembly and dynamic covalent chemistry. In contrast to the direct complexation of asymmetric ligand **L** with Pt(II) and Pd(II) ions, which can result in a large variety of possible products (up to 14 monomeric and 576 interlocked cages), the chosen subcomponent self‐assembly approach allowed us to obtain one PtPd**L**_4_ monomeric cage and one Pt_2_Pd_2_ **L**_8_ double cage as the major products, depending on the choice of solvent. In the structure of the interpenetrated dimer, the kinetically inert Pt(II) ions are located at the peripheral positions, while the more labile Pd(II) ions become buried inside the cage interior, sterically hindering their susceptibility to the attack of competing ligands. This configuration results in enhanced kinetic stability toward large amounts of competing ligands, unlike previously reported conventional Pd(II) cage catenanes. Furthermore, the strong affinity of the heterometallic cage catenane for halide anions enables it to participate in halide abstraction reactions from organic compounds. Our findings highlight the potential of heterometallic mechanically interlocked systems as a robust platform for applications in anion sensing and capture, halide abstraction, and anion‐binding catalysis.

## Supporting Information

The authors have cited additional references within the Supporting Information.^[^ ^60^, ^61^, ^62^, ^63^, ^64^, ^65^, ^66^, ^67^, ^68^, ^69^, ^70^, ^71^, ^72^, ^73^ ^]^ The X‐ray crystallographic coordinates for Pt_2_Pd_2_ **L**_8_ structure reported in this study have been deposited at the Cambridge Crystallographic Data Centre (CCDC), under deposition number 2453736. These data can be obtained free of charge from The Cambridge Crystallographic Data Centre via www.ccdc.cam.ac.uk/data_request/cif. The data that support the findings of this study are available from the corresponding author upon reasonable request.

## Conflict of Interests

The authors declare no conflict of interest.

## Supporting information



Supporting Information

Supporting Information

Supporting Information

## Data Availability

The data that support the findings of this study are available from the corresponding author upon reasonable request.
