# Supplementary material for: A Robust Heterometallic Pt2Pd2L8 Double Cage Catenane
Source: Angew Chem Int Ed Engl. 2025 Sep 30;64(47):e202516952. doi: 10.1002/anie.202516952 (PMC12624329; doi:10.1002/anie.202516952)
Supplement: Supplementary file 1 — Supporting Information [file ANIE-64-e202516952-s002.pdf]

A Robust Heterometallic $Pt_2Pd_2L_8$ Double Cage Catenane

Sudhakar Ganta,#[a] Alexander S. Mikherdov,#[a] Ananya Baksi,[a,b] Christoph Drechsler,[a] and Guido H. Clever*[a]

[a] Dr. S. Ganta, Dr. A. S. Mikherdov, Prof. Dr. A. Baksi, Dr. C. Drechsler, Prof. Dr. G. H. Clever
Department of Chemistry and Chemical Biology
TU Dortmund University
Otto-Hahn Straße 6, 44227 Dortmund, Germany
E-mail: guido.clever@tu-dortmund.de

[b] Prof. Dr. A. Baksi
Department of Chemistry
Jadavpur University
Raja S.C. Mallick Rd. 188, 700032, Kolkata, India

[#] These authors contributed equally to this work

Contents

S1 General procedures ... 2
S1.1 Materials and methods ... 2
S1.2 Experimental procedures ... 2
S2 Synthesis and characterization ... 3
S2.1 Ligand synthesis ... 3
S2.1.1 Synthesis of precursor B ... 3
S2.1.2 Synthesis of PTA ... 4
S2.1.3 Synthesis of L ... 6
S2.2 Statistical self-assembly of homometallic Pd(II) cage isomers ... 8
S2.3 Heterometallic cages self-assembly ... 10
S2.3.1 Statistical self-assembly of heterometallic Pt(II)-Pd(II) cage isomers ... 10
S2.3.2 Synthesis of $[Pt(PyCHO)_4(BF_4)_2]$ ... 14
S2.3.3 Self-assembly of heterometallic monomeric all-*syn* $PtPdL_4$ cage ... 15
S2.3.4 Self-assembly of heterometallic $Pt_2Pd_2L_8$ double cage ... 18
S3 Mechanistic study of double cage self-assembly ... 21
S3.1 Self-assembly of $Pd_n(PTA)_{2n}$ ... 21
S3.2 Monitoring of $Pt_2Pd_2L_8$ double cage assembly ... 23
S3.3 Possible mechanisms of $Pt_2Pd_2L_8$ double cages formation ... 25
S4 Halide uptake and cage stability study ... 28
S5 Halide abstraction studies ... 31
S6 X-ray crystallography ... 33
S7 Theoretical calculations ... 36
S8 References ... 44

S1 General procedures

S1.1 Materials and methods

NMR spectroscopic data were acquired using a Bruker AV 500 Avance NEO spectrometer. For ^{1}H NMR, chemical shifts were referenced to the solvent lock signal. Proton signals were assigned with the aid of 2D NMR spectra. ^{1}H DOSY NMR spectra were recorded using the dstebpgp3s pulse sequence, with diffusion delays (D20) ranging from 0.06 to 0.10 s and gradient pulse durations (P30) between 800 and 2000 µs. T_1 relaxation analyses of the corresponding signals in the 1D spectra were performed to determine diffusion coefficients (D) using the Stejskal–Tanner equation.[60, 61] Hydrodynamic radii r_H were calculated using the Stokes–Einstein equation.

Mass spectra and trapped ion mobilograms were recorded using a Bruker ESI timsTOF spectrometer in positive ion mode. For calibration, Agilent™ ESI Low Concentration Tuning Mix was used. The measured inverse mobility was converted to experimental collision cross section values ($^{TIMS}CCS_{N2}$) using the Mason-Schamp equation.[62]

S1.2 Experimental procedures

Where necessary, experiments were carried out under a nitrogen atmosphere using standard Schlenk techniques. Chemicals and standard solvents were purchased from Sigma-Aldrich, Acros Organics, Carl Roth, TCI Europe, VWR, and ABCR, and were used as received unless stated otherwise. Dry solvents were either purchased or purified and dried over absorbent-filled columns using a GS-Systems solvent purification system (SPS).

Reactions were monitored by thin-layer chromatography (TLC) using silica-coated aluminum plates (Merck, silica 60, fluorescence indicator F254, thickness 0.25 mm). Column chromatography was performed using silica gel (Merck, silica 60, 0.02–0.063 mm, mesh ASTM) as the stationary phase. Recycling gel permeation chromatography

(GPC) was conducted on Japan Analytical Industry NEXT and LaboACE instruments, equipped with JAIGEL 1-HH and 2-HH columns (20 mm × 600 mm).

S2 Synthesis and characterization

S2.1 Ligand synthesis

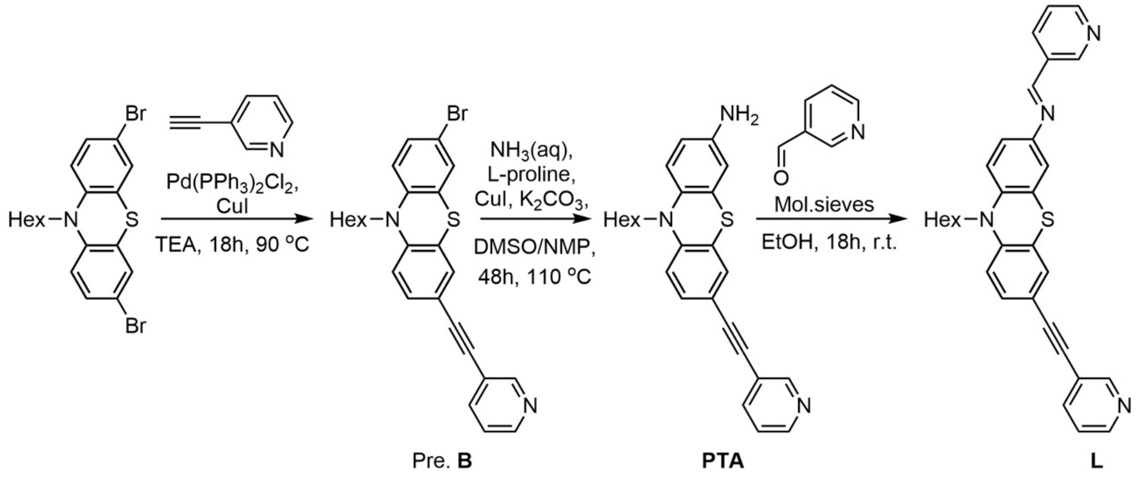

Scheme S1. Synthesis of **PTA** and **L**.

S2.1.1 Synthesis of precursor B

A mixture of 3,7-dibromo-10-hexyl-10H-phenothiazine[63] (1 g, 2.3 mmol, 1 eq.), 3-ethynylpyridine (235 mg, 2.3 mmol, 1 eq.), and CuI (45 mg, 0.23 mmol, 0.1 eq.) was dissolved in triethylamine (15 mL) and degassed for 30 min. $Pd(PPh_3)_2Cl_2$ (77 mg, 0.12 mmol, 0.05 eq.) was then added, and the reaction mixture was stirred at 90 °C for 18 h. After the reaction completion, the solvent was removed under reduced pressure. Ethyl acetate (25 mL) and aqueous ammonia (10 mL) were added to the residue. The organic phase was washed with water (3 × 10 mL), dried over $MgSO_4$, and filtered. After evaporation of the solvent under reduced pressure, a highly viscous liquid was obtained. The crude product was purified by column chromatography on silica gel (pentane : ethyl acetate = 3:1) to yield pure precursor B (690 mg, 65% yield).

^{1}H NMR (500 MHz, CDCl3): δ [ppm] = 8.37 (d, J = 2.5 Hz, 1 H), 8.21 (dd, J = 4.9 Hz, 1 H), 7.51 (dt, J = 7.9 Hz, 1 H), 7.06-6.96 (m, 5 H), 6.65 (d, J = 8.4 Hz, 1 H), 6.55 (d, J = 8.4

Hz, 1 H), 3.54 (t, J = 7.0 Hz, 2 H), 1.14 (qui, J = 7.6 Hz, 2 H), 1.09 (qui, J = 8.3 Hz, 2 H), 0.97 – 0.94 (m, 4 H), 0.53 (t, J = 7.1 Hz, 3 H).

ESI-HRMS $[C_{25}H_{23}N_2SBr + H]^+$: found: 465.0725; calc.: 465.0817

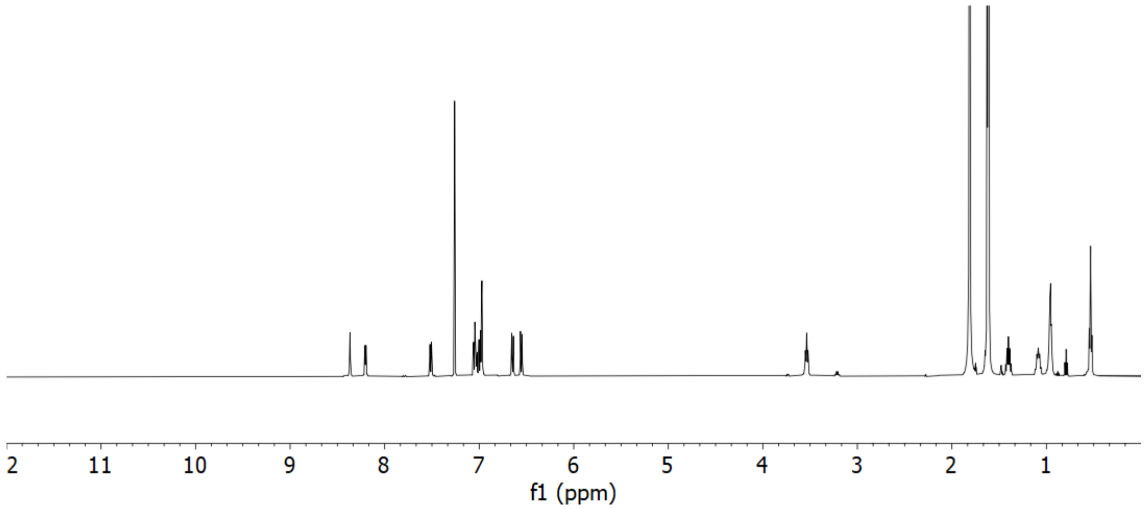

Figure S1. ^{1}H NMR (500 MHz, 298 K, $CDCl_3$) spectrum of precursor **B.**

S2.1.2 Synthesis of PTA

Precursor **B** (650 mg, 1.39 mmol, 1 eq.), L-proline (65 mg, 0.56 mmol, 0.4 eq.), CuI (53 mg, 0.28 mmol, 0.2 eq.), and K_2CO_3 (580 mg, 4.2 mmol, 3 eq.) were dissolved in a mixture of DMSO (2.5 mL) and N-methyl-2-pyrrolidone (2.5 mL), and the solution was degassed for 30 min. A 35% aqueous ammonia solution (6 mL) was then added, and the reaction mixture was stirred at 110 °C for 48 h. After the reaction completion, the reaction mixture was cooled to room temperature, water (10 mL) was added, and the product was extracted with ethyl acetate (3 × 15 mL). The combined organic layers were washed with water (10 mL), dried over $MgSO_4$, and filtered. Evaporation of the solvent under reduced pressure yielded a highly viscous liquid. The crude product was purified by column chromatography on silica gel (pentane : ethyl acetate = 3:1), followed by gel permeation chromatography (GPC), to afford **PTA** as the pale yellow solid (390 mg, 70% yield).

^{1}H NMR (500 MHz, CDCl3): δ [ppm] = 8.72 (d, J = 1.5 Hz, 1 H), 8.51 (dd, J = 4.8 Hz, 1 H), 7.78 (dt, J = 7.9 Hz, 1 H), 7.30-7.26 (m, 3 H), 6.80-6.72 (b, 1 H), 6.67 (d, J = 8.8 Hz, 1 H), 6.55 (b, 2 H), 3.86-3.67 (b, 2 H), 1.76 (quin., J = 6.8 Hz, 2 H), 1.41 (q, J = 6.8 Hz, 2 H), 1.34-1.29 (m, 4 H), 0.87 (t, J = 6.8 Hz, 3 H).

^{13}C NMR (126 MHz, 298 K, CDCl3) δ [ppm] = 151.7, 147.8, 146.6, 139.9, 137.5, 131.1, 130.3, 124.4, 126.08, 123.3, 121.2, 116.4,115.5,115.4,115.3, 114.8, 92.9, 85.5, 55.6, 47.8, 31.6, 26.7, 22.7, 14.1.

ESI-HRMS $[C_{25}H_{25}N_3S+H]^+$: found: 400.1815; calc.: 400.1842

^{1}H NMR (500 MHz, DMSO-*d*6): δ [ppm] = 8.71 (d, J = 1.6 Hz, H_g, 1 H), 8.55 (dd, J = 4.9 Hz, H_f, 1 H), 7.93 (dd, J = 8.1 Hz, H_d, 1 H), 7.45 (dd, J = 8.4 Hz, H_e, 1 H), 7.34 (dd, J = 8.4 Hz, H_c, 1 H), 7.28 (d, J = 1.8 Hz, H_a, 1 H), 6.94 (d, J = 8.6 Hz, H_b, 1 H), 6.75 (d, J = 8.6 Hz, $H_{c'}$, 1 H), 6.44 (d, J = 8.6 Hz, $H_{b'}$, 1 H), 6.40 (d, J = 2.2 Hz, $H_{a'}$, 1 H), 4.91 (b, NH_2, 2 H), 3.78 (d, J = 6.9 Hz, N-CH_2, 2 H), 1.65 (qui, J = 7.0 Hz, CH_2, 2 H), 1.37 (qui, J = 6.9 Hz, CH_2, 2 H), 1.30 – 1.21 (m, CH_2, 4 H), 0.84 (t, J = 6.9 Hz, CH_3, 3 H).

^{1}H NMR (500 MHz, CD_3CN): δ [ppm] = 8.71 (d, J = 1.6 Hz, H_g, 1 H), 8.54 (dd, J = 4.7 Hz, H_f, 1 H), 7.85 (d, J = 8.3 Hz, H_d, 1 H), 7.38 – 7.34 (m, H_e, H_c, 2 H), 7.29 (s, H_a, 1 H), 6.91 (b, H_b, 1 H), 6.77 (dd, J = 7.4 Hz, $H_{c'}$, 1 H), 6.58 – 6.51 (b, $H_{b'}$, 1 H), 6.50 (d, J = 1.8 Hz, $H_{a'}$, 1 H), 3.98 – 3.83 (b, NH_2 and N-CH_2 4 H), 1.73 (qui, J = 7.5 Hz, CH_2, 2 H), 1.43 (qui, J = 7.3 Hz, CH_2, 2 H), 1.32 – 1.22 (m, CH_2, 4 H), 0.89 (t, J = 7.2 Hz, CH_3, 3 H).

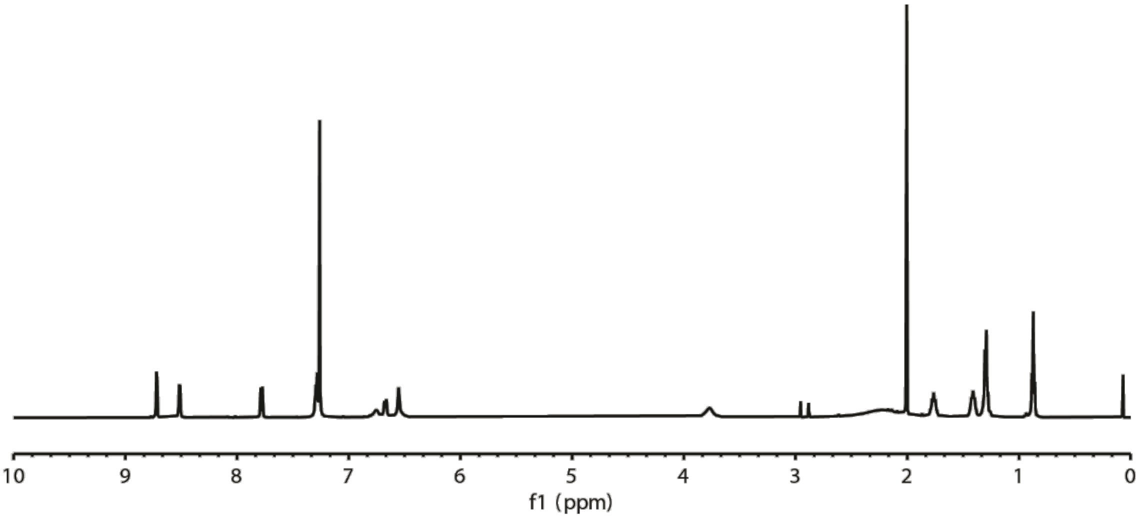

Figure S2. ^{1}H NMR (500 MHz, 298 K, $CDCl_3$) spectrum of **PTA**.

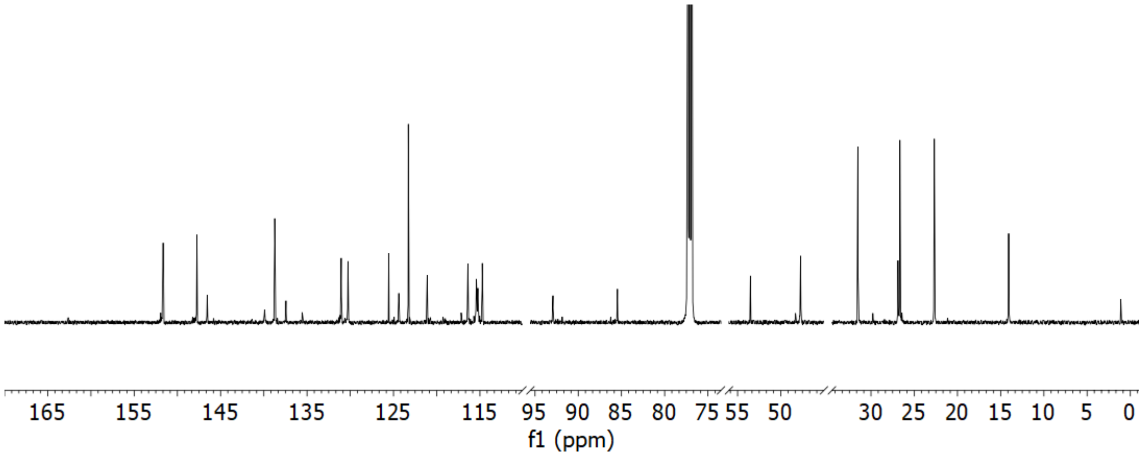

Figure S3: ^{13}C NMR (126 MHz, 298 K, $CDCl_3$) spectrum of **PTA**.

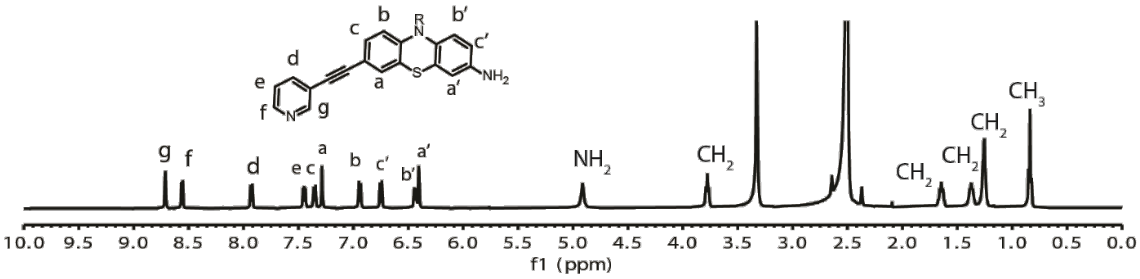

Figure S4. ^{1}H NMR (500 MHz, DMSO-d_6) spectrum of **PTA**.

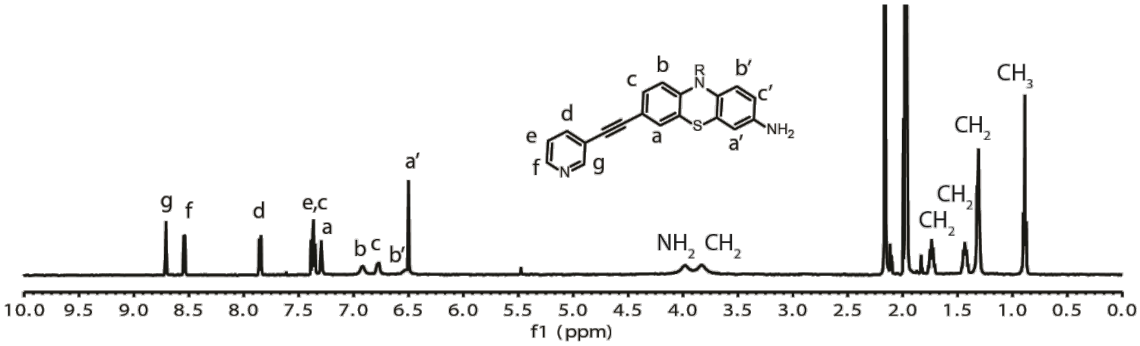

Figure S5. ^{1}H NMR (500 MHz, CD_3CN) spectrum of **PTA**.

S2.1.3 Synthesis of L

A mixture of PTA (13 mg, 0.0323 mmol, 1 eq.) and **PyCHO** (nicotinaldehyde) (6 mg, 0.0485 mmol, 1.5 eq.) was dissolved in ethanol (0.5 mL) in the presence of molecular

sieves. The solution was stirred at room temperature for 18 h. It was then filtered, and the solvent was evaporated under vacuum to yield a highly viscous liquid. The final product was recrystallized from toluene to afford 11 mg of ligand **L** (75% yield).

^{1}H NMR (500 MHz, CD3CN): δ [ppm] = 9.03 (d, J = 1.7 Hz, $H_{g'}$, 1 H), 8.72 (dd, J = 2.2 Hz, H_g, 1 H), 8.69 (dd, J = 4.8 Hz, $H_{f'}$, 1 H), 8.65 (s, CH, 1 H), 8.55 (dd, J = 4.8 Hz, H_f, 1 H), 8.27 (dt, J = 4.9 Hz, $H_{d'}$, 1 H), 7.87 (dt, J = 7.8 Hz, H_d, 1 H), 7.49 (dd, J = 7.9 Hz, $H_{e'}$, 1 H), 7.42-7.37 (m, H_c, H_e, 2H), 7.35 (d, J = 2.1 Hz, H_a, 1 H), 7.31 (dd, J = 8.5 Hz, $H_{c'}$, 1 H), 7.18 (d, J = 2.5 Hz, $H_{a'}$, 1 H), 7.06 (d, J = 8.8 Hz, $H_{b'}$, 1 H), 7.01 (d, J = 8.7 Hz, H_b, 1 H), 3.95 (t, J = 7.0 Hz, N-CH_2, 2 H), 1.47 (qui, J = 8.3 Hz, CH_2, 2 H), 1.35 – 1.30 (m, CH_2, 6 H), 0.89 (t, J = 7.1 Hz, CH_3, 3 H).

ESI-HRMS $[C_{31}H_{28}N_4S + H]^+$: found: 489.2132; calc.: 489.2107

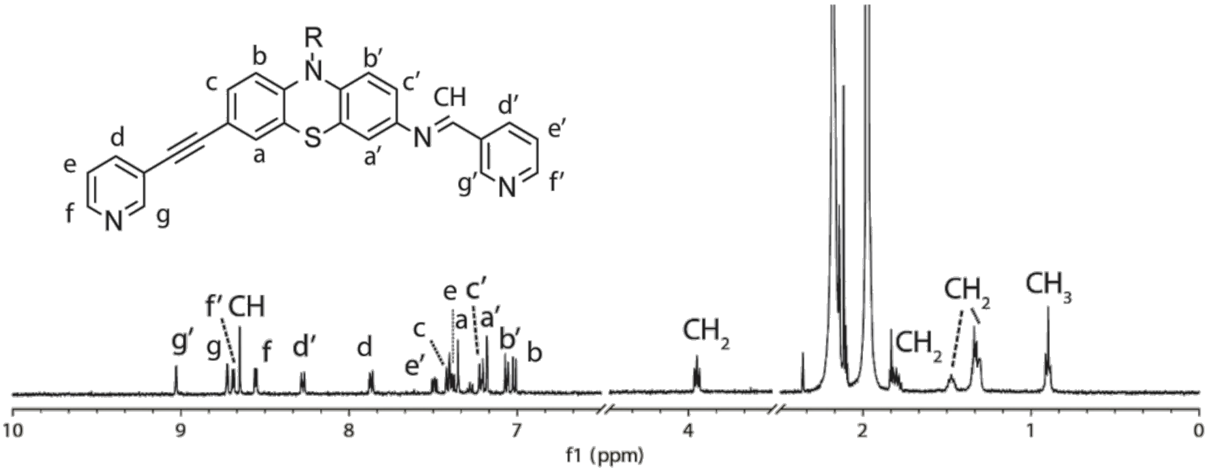

Figure S6. ^{1}H NMR (500 MHz, CD_3CN) spectrum of ligand **L**.

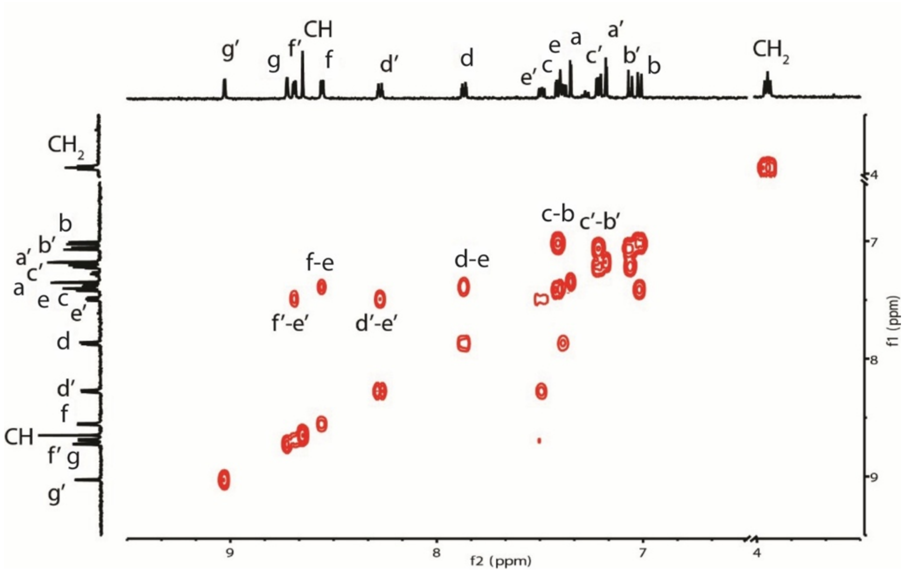

Figure S7. Partial ^{1}H-^{1}H COSY (500 MHz, CD_3CN) spectrum of ligand **L**.

S2.2 Statistical self-assembly of homometallic Pd(II) cage isomers

The asymmetric ligand L features a tricyclic backbone connected to pyridine donors via one ethynyl and one imine linker, which differ slightly in length (4.0 Å vs 3.7 Å) and geometry. As a result, direct complexation of L with Pd(II) ions can lead to the formation of four possible isomeric Pd_2L_4 cages with different ligand orientations: ***trans-2syn-2anti*** (***D*$_{2d}$** symmetry), ***cis-2syn-2anti*** (***C*$_{2h}$**), 3syn-1anti (***C*$_s$**), and all-syn (*C*$_{4v}$) isomers (Scheme S2). Upon catenation, these four Pd_2L_4 cages can form four ***homo***-interlocked and six ***hetero***-interlocked pairs. Depending on the symmetry of the monomeric cages, each pair can produce between two and twelve orientational isomers (including enantiomers), yielding a total of 42 isomeric Pd_4L_8 double cages (21 ***homo***-interlocked and 21 ***hetero***-interlocked; Scheme S2).

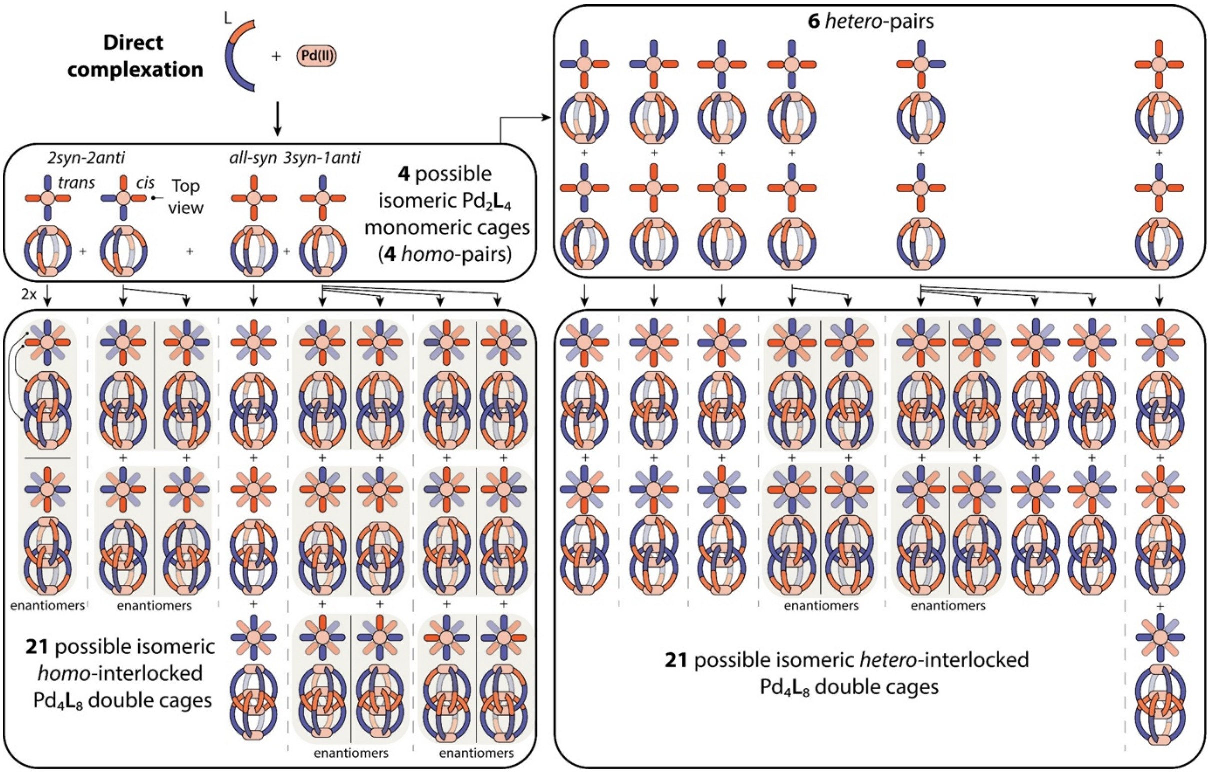

Scheme S2. Possible monomeric cage products of the direct complexation of Pd(II) ions with asymmetric ligand **L** and their catenation to homo- and hetero-interlocked Pd_4**L**$_8$ double cages (same number of isomers is possible for Pt_4**L**$_8$ species).

A mixture of homometallic Pd(II) cages was prepared by the reaction of assymetric ligand **L** (1.4 µmol, 140 µL of a 10 mM solution in CD_3CN) with $[Pd(CH_3CN)_4(BF_4)_2]$ (0.70 µmol, 70 µL of a 10 mM solution in CD_3CN) in 300 µL of CD_3CN at 70 °C for 24 hours. The multiple broadened signals observed in the ^1H NMR spectrum (**Figure S8**) suggest the formation of a complex mixture of cages. ESI-MS analysis reveals $[3BF_4@Pd_4$**L**$_8]^{5+}$ as the dominant ion (**Figure S9**), which, according to TIMS measurements, exhibits a single and broader CCS distribution (FWHH ~ 6.5Å) than the ones observed for $[3BF_4@Pt_2Pd_2$**L**$_8]^{5+}$ ions (FWHH ~ 2.5–5 Å).

These findings support the hypothesis that the complexation of **L** with Pd(II) ions in CD_3CN results in a mixture of isomeric double cages, likely involving various orientations of ligand **L** within the cage structure (**Scheme S2**).

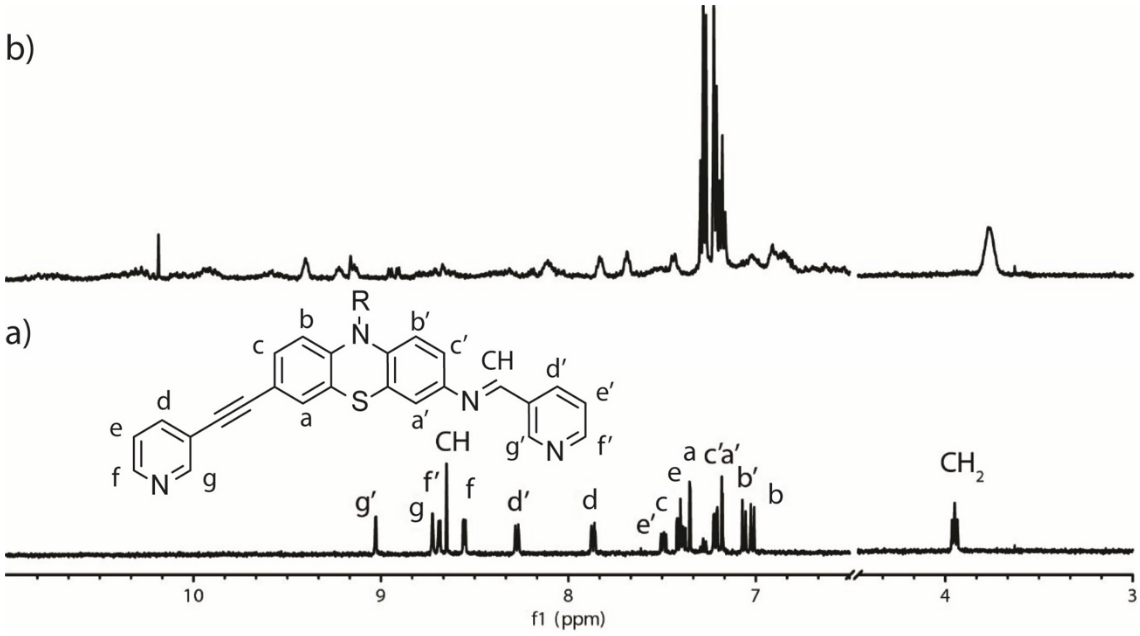

Figure S8. ^{1}H NMR (500 MHz, CD_3CN) spectra of free ligand **L** (a) and its assembly with Pd(II) ions (b).

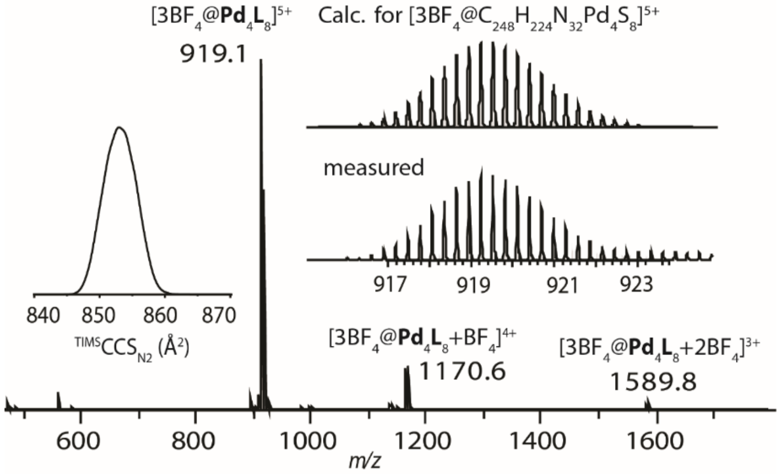

Figure S9. ESI-MS of Pd(II) assembly of **L** with inset showing a broad ion mobility trace for the [3BF4@Pd$_4$**L**$_8$]$^{5+}$ ion.

S2.3 Heterometallic cages self-assembly

S2.3.1 Statistical self-assembly of heterometallic Pt(II)-Pd(II) cage isomers

Similarly to the complexation with only Pd(II) ions, the direct reaction of ligand **L** with a mixture of Pd(II) and Pt(II) ions is not expected to proceed under strict geometrical

control and would likely result in an even greater variety of products (not considering any kinetic biases for the formation of Pd vs. Pt species): four homometallic Pd_2**L**$_4$ and four homometallic Pt_2**L**$_4$ cages, along with six heterometallic PtPd**L**$_4$ cages. Upon catenation, these 14 cages may form 105 possible pairs (14 *homo*-interlocked and 91 *hetero*-interlocked). Depending on the symmetry of the monomeric cages, each pair can produce between two and sixteen orientational isomers, leading to a total of 576 potential quadruply interlocked cage products. The first group includes 42 homometallic Pd_4**L**$_8$ and 42 homometallic Pt_4**L**$_8$ double cages, formed from catenation of the respective homometallic M_2**L**$_4$ cages (M = Pd or Pt, see Scheme S2). The second group comprises 136 possible heterometallic $PtPd_3$**L**$_8$ and 136 Pt_3Pd**L**$_8$ cages, formed through the interaction of the six heterometallic PtPd**L**$_4$ cages with the four homometallic M_2**L**$_4$ (M = Pd or Pt) cages (see Scheme S3). The third group consists of 220 possible Pt_2Pd_2**L**$_8$ structures. These arise either from interactions among the six PtPd**L**$_4$ cages (yielding 42 *homo*-interlocked and 108 *hetero*-interlocked structures, Scheme S4) and from combinations between four homometallic Pd_2**L**$_4$ and four homometallic Pt_2**L**$_4$ cages (yielding 70 *hetero*-interlocked structures, Scheme S5).

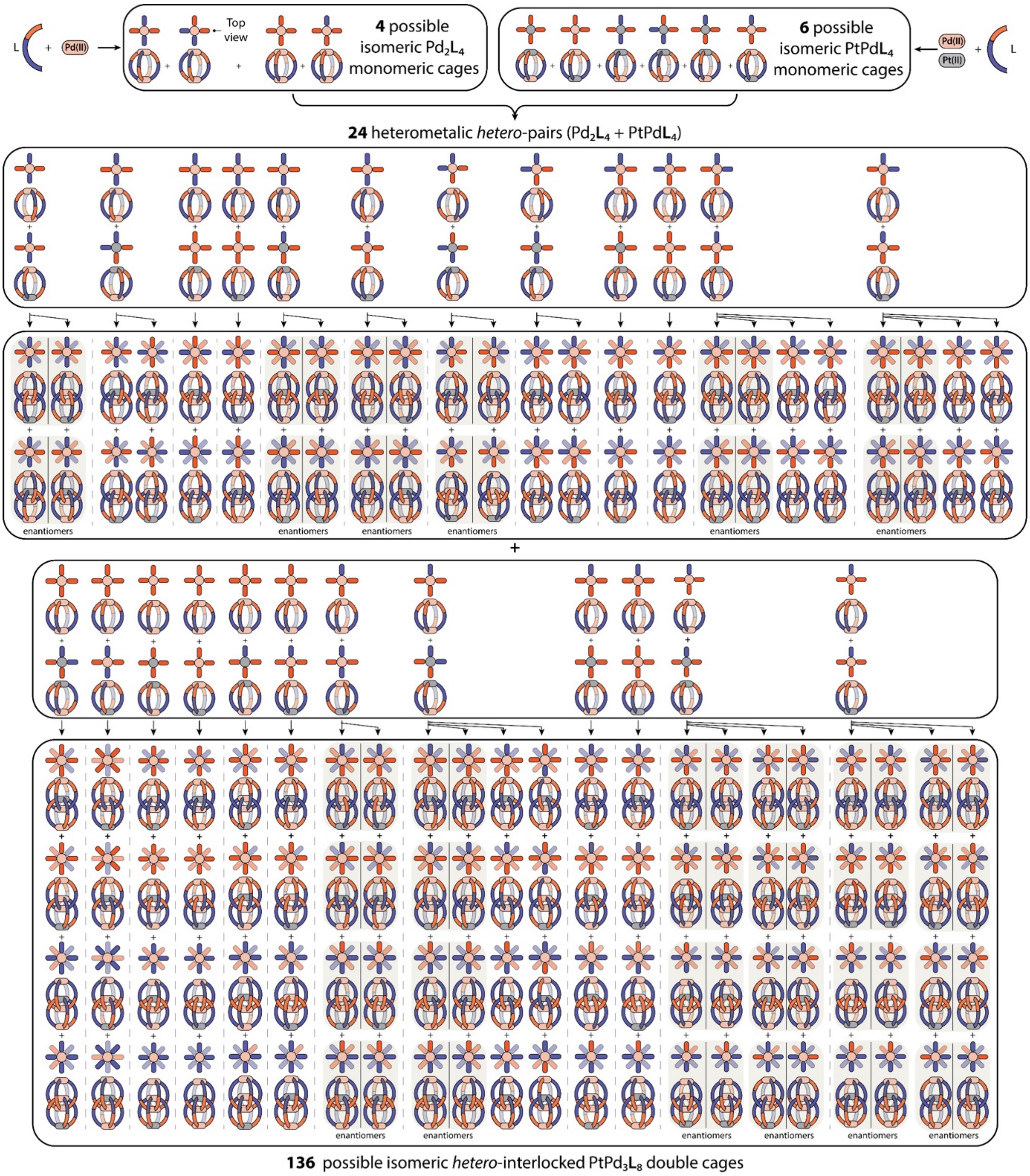

Scheme S3. Possible cage products of direct complexation of Pd(II) and Pt(II) ions with asymmetric ligand **L** and their catenation to *hetero*-interlocked $PtPd_3\mathbf{L}_8$ double cages (same number of isomers is possible for $Pt_3Pd\mathbf{L}_8$ species).

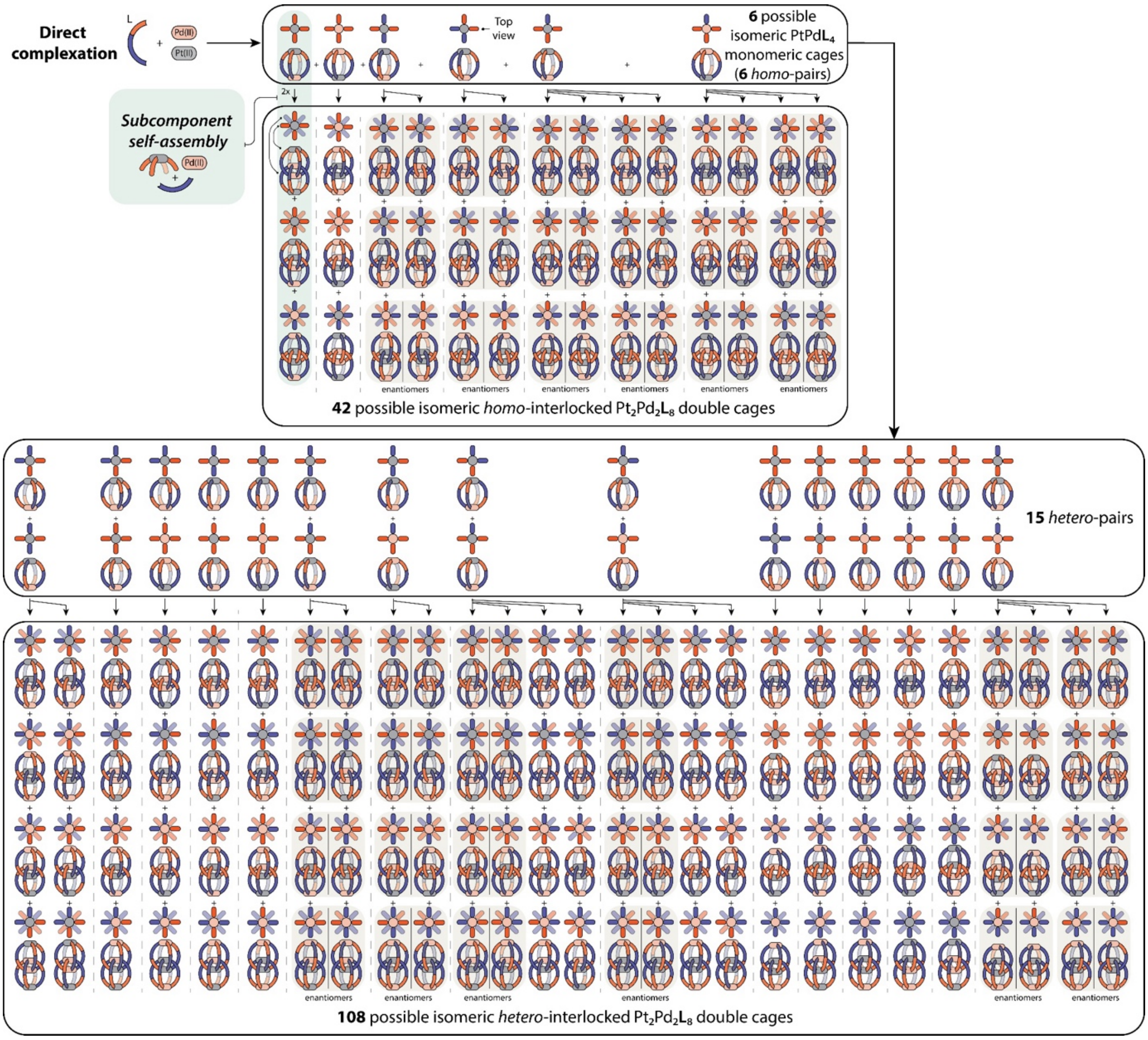

Scheme S4. Possible cage products of direct complexation of Pd(II) and Pt(II) ions with asymmetric ligand **L** and their catenation to *homo*- and *hetero*-interlocked $Pt_2Pd_2\mathbf{L}_8$ double cages (not considering any kinetic biases for the formation of Pd vs. Pt species).

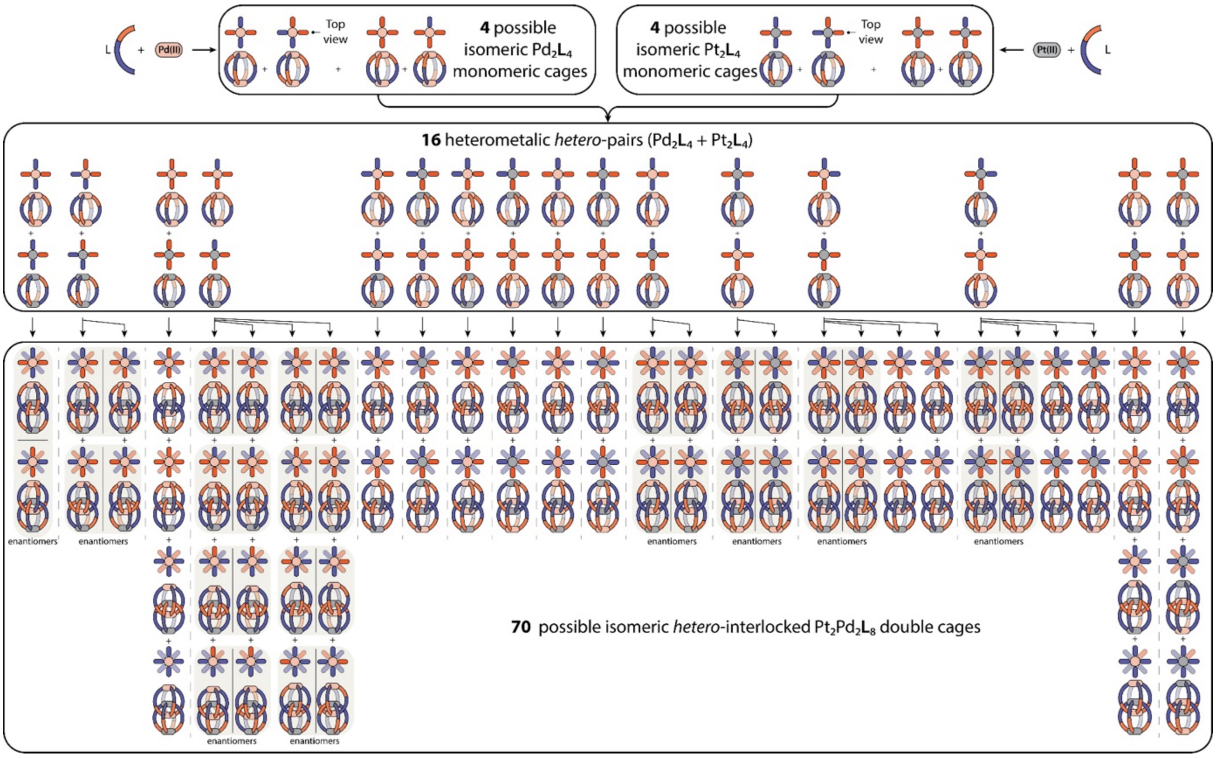

Scheme S5. Possible cage products of direct complexation of Pd(II) and Pt(II) ions with asymmetric ligand **L** and their catenation to *hetero*-interlocked $Pt_2Pd_2\mathbf{L}_8$ double cages.

S2.3.2 Synthesis of [Pt(PyCHO)$_4$(BF$_4$)$_2$]

[Pt(**PyCHO**)$_4$(BF$_4$)$_2$] was synthesized according to the literature procedure.[28, main tex]

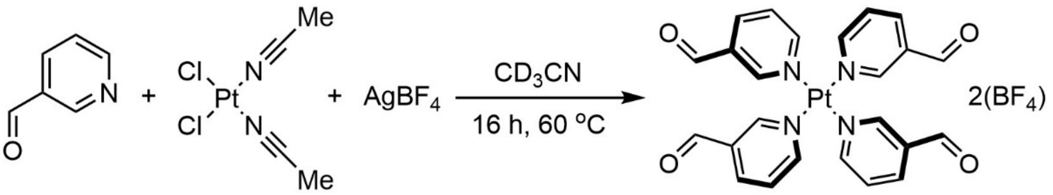

Scheme S6. Synthesis of [Pt(**PyCHO**)$_4$(BF$_4$)$_2$].

^{1}H NMR (500 MHz, CD_3CN): δ [ppm] = 10.04 (s, CHO, 4 H), 9.37 (s, $H_{g'}$, 4 H), 9.13 (d, J = 5.6 Hz, $H_{f'}$, 4 H), 8.41 (d, J = 7.9 Hz, $H_{d'}$, 4 H), 7.78 (dd, J = 7.7 Hz, $H_{e'}$, 4 H).

^{1}H NMR (500 MHz, DMSO-*d6*): δ [ppm] = 10.00 (s, CHO, 4 H), 9.66 (s, $H_{g'}$, 4 H), 9.38 (d, J = 5.8 Hz, $H_{f'}$, 4 H), 8.48 (d, J = 7.9 Hz, $H_{d'}$, 4 H), 7.89 (dd, J = 7.8 Hz, $H_{e'}$, 4 H).

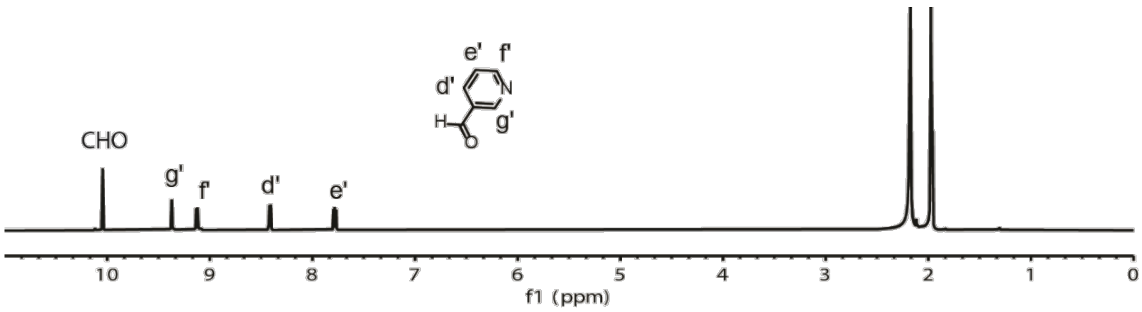

Figure S10. ^{1}H NMR (500 MHz, CD_3CN) spectrum of [Pt(**PyCHO**)$_4$(BF_4)$_2$].

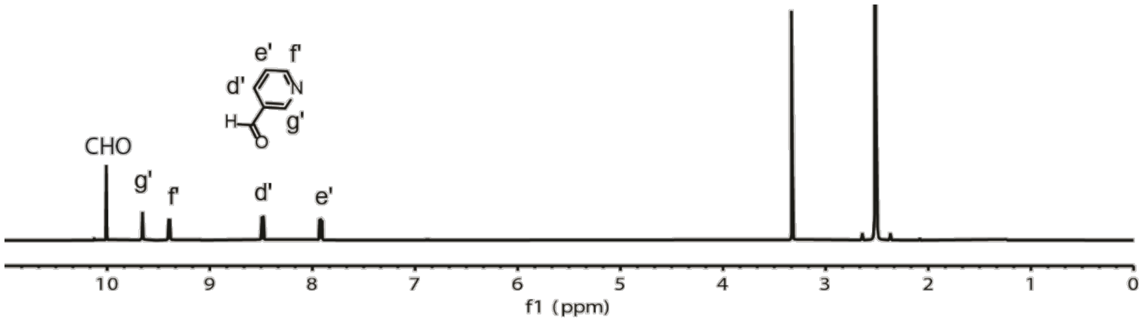

Figure S11. ^{1}H NMR (500 MHz, DMSO-d_6) spectrum of [Pt(**PyCHO**)$_4$(BF_4)$_2$].

S2.3.3 Self-assembly of heterometallic monomeric all-*syn* PtPdL_4 cage

A single isomer of the heterometallic monomeric cage was prepared by the reaction of **PTA** (1.4 µmol, 74 µL of a 20 mM solution in DMSO-d_6), [Pt(**PyCHO**)$_4$(BF_4)$_2$] (0.40 µmol, 40 µL of a 10 mM solution in DMSO-d_6) and [Pd(CH_3CN)$_4$(BF_4)$_2$] (0.35 µmol, 35 µL of a 10 mM solution in DMSO-d_6) in 350 µL of DMSO-d_6 at 30 °C for 24 h to give a 0.7 mM solution of all-*syn* PtPd**L**$_4$.

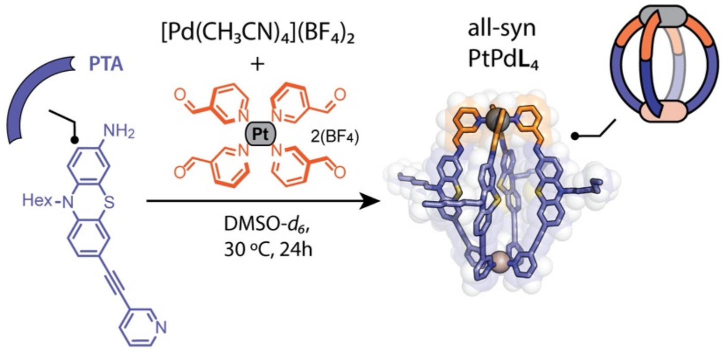

Scheme S7. Self-assembly of heterometallic monomeric cage PtPd**L**$_4$

^{1}H NMR (500 MHz, DMSO-*d6*): δ [ppm] = 9.91 (s, $H_{g'}$, 1 H), 9.50 (d, J = 1.8 Hz, H_g, 1 H), 9.41 (d, J = 5.1 Hz, $H_{f'}$, 1 H), 9.25 (d, J = 5.2 Hz, H_f, 1 H), 8.68 (s, N=CH,1 H), 8.43 (d, J = 8.1 Hz, $H_{d'}$, 1 H), 8.19 (d, J = 8.1 Hz, H_d,1 H), 7.89 (dd, J = 6.4 Hz, $H_{e'}$, 1 H), 7.77 (d, J = 5.8 Hz, H_e, 1 H), 7.44 – 7.40 (b, H_c, H_a,2 H), 7.27-7.22 (b, $H_{c'}$, $H_{a'}$, 2 H), 7.09 (t, J = 8.8 Hz, $H_{b'}$, H_b, 2 H), 3.91 (t, J = 7.1 Hz, N-CH_2, 2 H), 1.66 (qui, J = 7.0 Hz, CH_2, 2 H), 1.37 (qui, J = 7.3 Hz, CH_2, 2 H), 1.30 – 1.21 (m, CH_2, 4 H), 0.78 (t, J = 6.9 Hz, CH_3, 3 H).

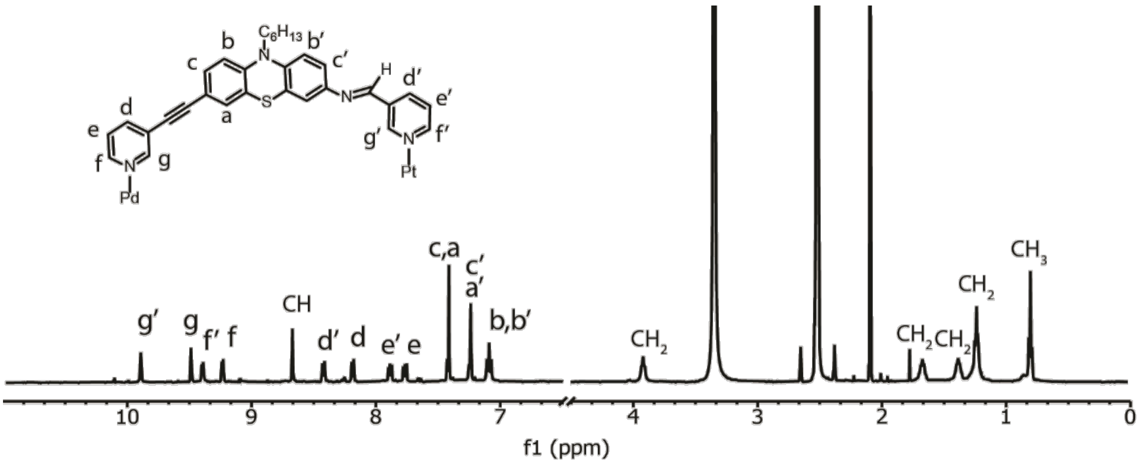

Figure S12. ^{1}H NMR (500 MHz, DMSO-d_6) spectrum of PtPd**L**$_4$.

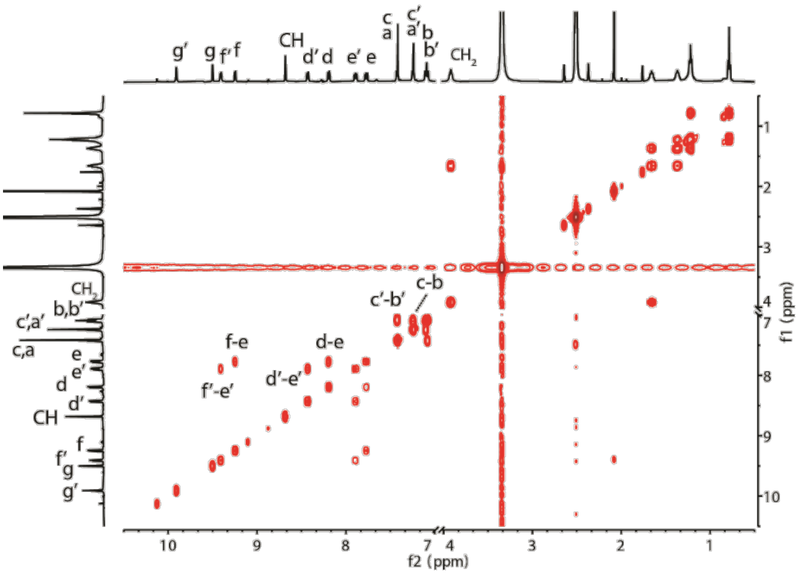

Figure S13.^{1}H-^{1}H COSY (500 MHz, DMSO-d_6) spectrum of PtPd**L**$_4$.

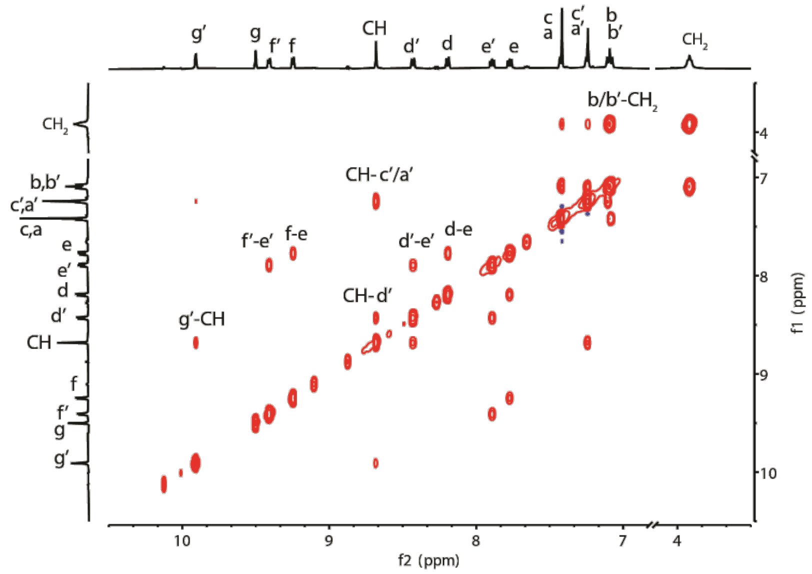

Figure S14. Partial ^{1}H-^{1}H NOESY (500 MHz, DMSO-d_6) spectrum of PtPd$\mathbf{L}_4$.

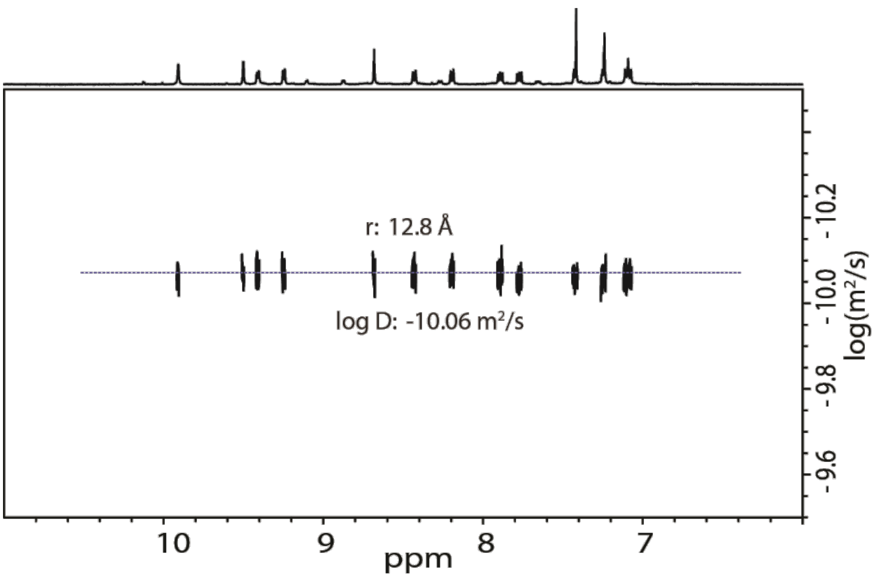

Figure S15. ^{1}H DOSY (500 MHz, 298K, DMSO-d_6) spectrum of PtPd$\mathbf{L}_4$ (D = 8.54×10^{-11} m^2s^{-1}; logD = –10.06 m^2s^{-1}; r_H = 12.8 Å).

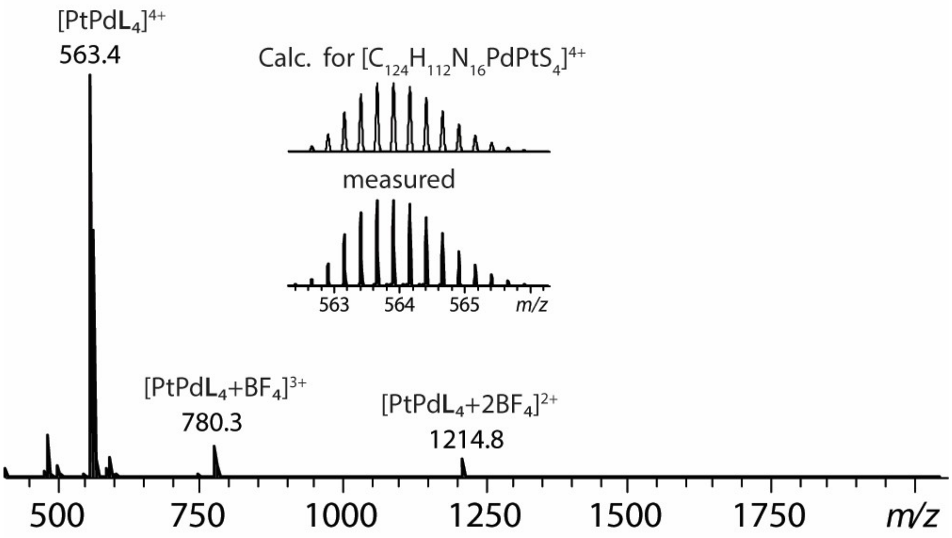

Figure S16. ESI-MS spectrum of PtPd**L**$_4$.

S2.3.4 Self-assembly of heterometallic $Pt_2Pd_2L_8$ double cage

Heterometallic double cage Pt_2Pd_2**L**$_8$ (one major isomer and two minor isomers) was prepared by the reaction of **PTA** (1.4 µmol, 74 µL of a 20 mM solution in CD_3CN), [Pt(**PyCHO**)$_4$(BF_4)$_2$] (0.40 µmol, 40 µL of a 10 mM solution in CD_3CN) and [Pd(CH_3CN)$_4$(BF_4)$_2$] (0.35 µmol, 35 µL of a 10 mM solution in CD_3CN) in 350 µL of CD_3CN at 30 °C for 48 h to give a 0.35 mM, 05 mL solution of Pt_2Pd_2**L**$_8$.

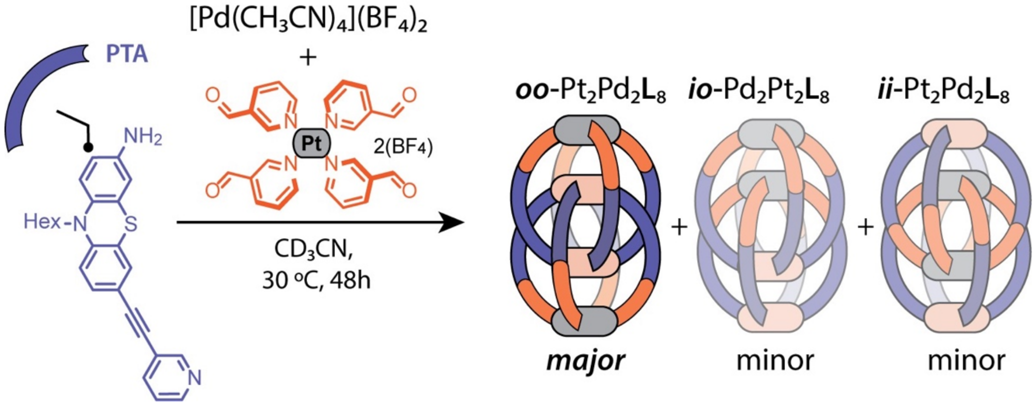

Scheme S8. Self-assembly of heterometallic double cages Pt_2Pd_2**L**$_8$.

^{1}H NMR (500 MHz, CD_3CN, only major isomer): δ [ppm] = 10.27 (b, $H_{g'}$, H_g, 2 H), 9.57 (d, J = 5.1 Hz, H_f,1 H), 9.31 (d, J = 5.9 Hz, $H_{f'}$, 1 H), 8.72 (s, N=CH, 1 H), 8.38 (d, J = 8.3 Hz, $H_{d'}$, 1 H), 7.80 (dd, J = 5.9 Hz, $H_{e'}$, 1 H), 7.27 – 7.24 (m, H_d, H_c, $H_{c'}$, 3 H), 7.01 (d, J = 8.7 Hz, $H_{b'}$, 3 H), 6.87 (d, J = 8.5 Hz, H_b, 1 H), 6.73 (d, J = 1.9 Hz, H_a,1H), 6.61– 6.59 (b, H_e, $H_{a'}$, 2 H), 3.79 (d, J = 6.8 Hz, N-CH_2,2H), 1.62 (qui, J = 7.9 Hz, CH_2, 2 H), 1.36 – 1.13 (m, CH_2, 6 H), 0.78 (t, J = 7.9 Hz, CH_3, 3 H).

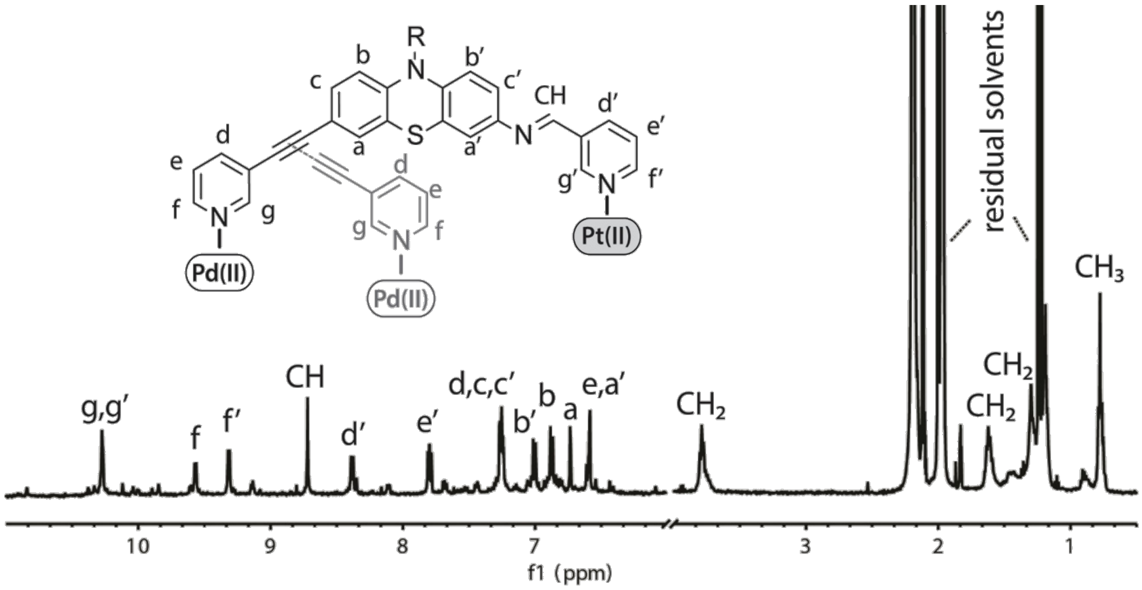

Figure S17. ^{1}H NMR (500 MHz, CD_3CN) spectrum of $Pt_2Pd_2\mathbf{L}_8$.

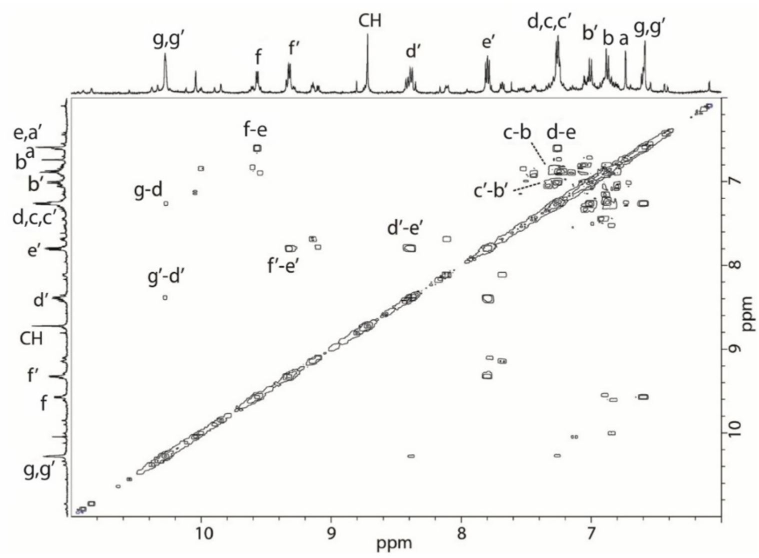

Figure S18. Partial ^{1}H–^{1}H COSY (500 MHz, CD_3CN) spectrum of $Pt_2Pd_2\mathbf{L}_8$.

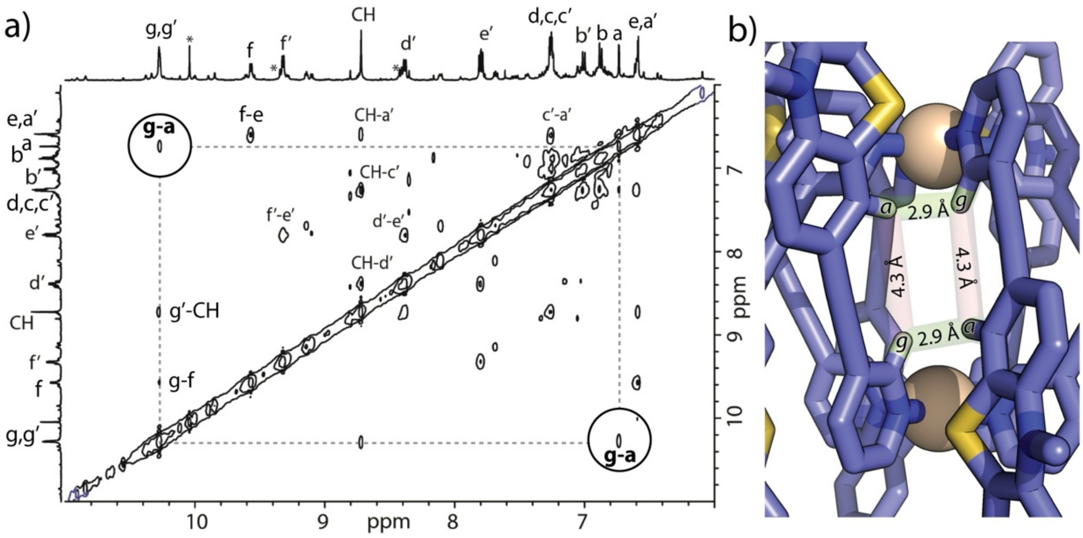

Figure S19. (a) Partial ^1H–^1H NOESY (500 MHz, CD_3CN) spectrum of $Pt_2Pd_2$$\mathbf{L}_8$ and (b) illustration of short contacts between protons ***g*** and ***a*** in the *oo*-$Pt_2Pd_2$$\mathbf{L}_8$ structure.

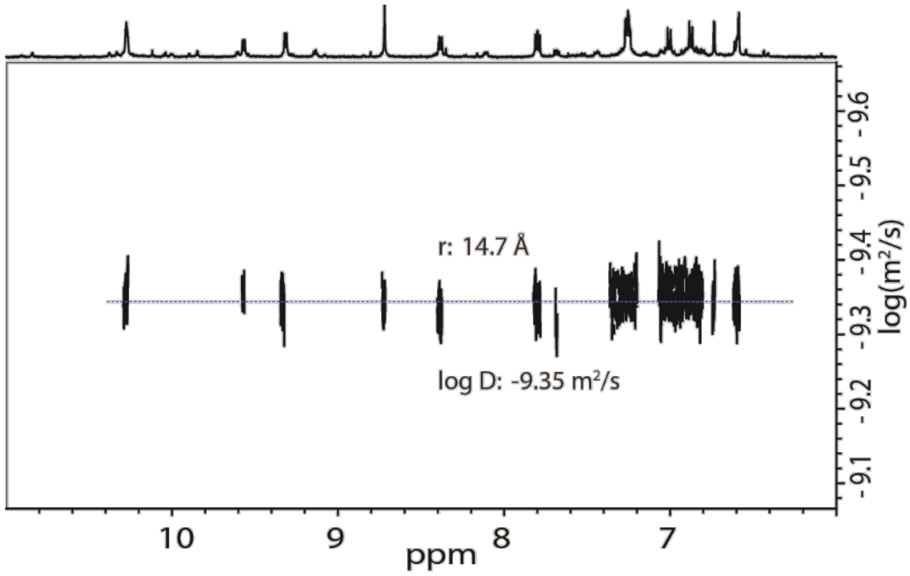

Figure S20. ^1H DOSY (500 MHz, 298K, CD_3CN) spectrum of $Pt_2Pd_2$$\mathbf{L}_8$ ($D = 4.43\times10^{-10}$ m^2s^{-1}, log D = -9.35 m^2s^{-1}, r_H = 14.7 Å).

S3 Mechanistic study of double cage self-assembly

S3.1 Self-assembly of $Pd_n(PTA)_{2n}$

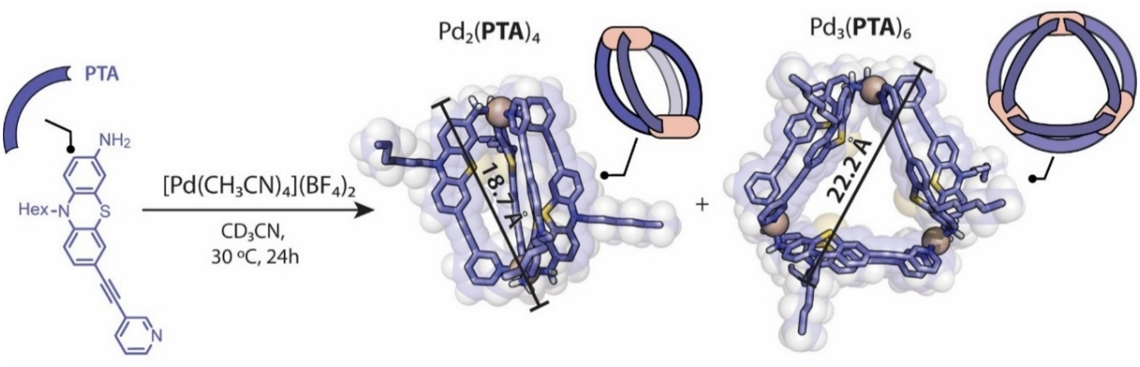

Scheme S9. Self-assembly of **PTA** with Pd(II) ions.

The reaction between **PTA** and Pd(II) ions was studied. Solutions of **PTA** (1.4 µmol, 74 µL of a 20 mM solution in CD_3CN) and $[Pd(CH_3CN)_4(BF_4)_2]$ (0.70 µmol, 70 µL of a 10 mM solution in CD_3CN) were combined, diluted with 360 µL of CD_3CN, and left at 30 °C for 24 hours to afford a mixture of Pd_n(**PTA**)$_{2n}$ species (n = 2, 3; **Scheme S9**).

The formation of these Pd(II) complexes was monitored by ^{1}H NMR spectroscopy and further characterized by high-resolution ESI-MS and ^{1}H DOSY NMR. After 30 minutes, the ^{1}H NMR spectrum revealed two sets of new signals compared to the free ligand, each with similar intensity (**Figure S21**). After 24 hours, the intensity of one set decreased relative to the other, suggesting a dynamic equilibrium between species.

High-resolution ESI-MS detected ions corresponding to Pd_2(**PTA**)$_4$ or Pd_3(**PTA**)$_6$ (**Figure S22**), and DOSY NMR analysis supported these findings by showing two distinct diffusion coefficients (**Figure S23**). The absence of signal splitting in the ^{1}H NMR spectra of Pd_2(**PTA**)$_4$ or Pd_3(**PTA**)$_6$ indicates symmetric structures, where each Pd(II) center is likely coordinated to two amino and two pyridine groups in a *cis*-arrangement.[38,main text]

The hydrodynamic radii obtained from the DOSY measurements were consistent with those predicted for the proposed molecular models, *cis*-Pd_2(**PTA**)$_4$ or *cis*-Pd_3(**PTA**)$_6$ (**Scheme S9**).

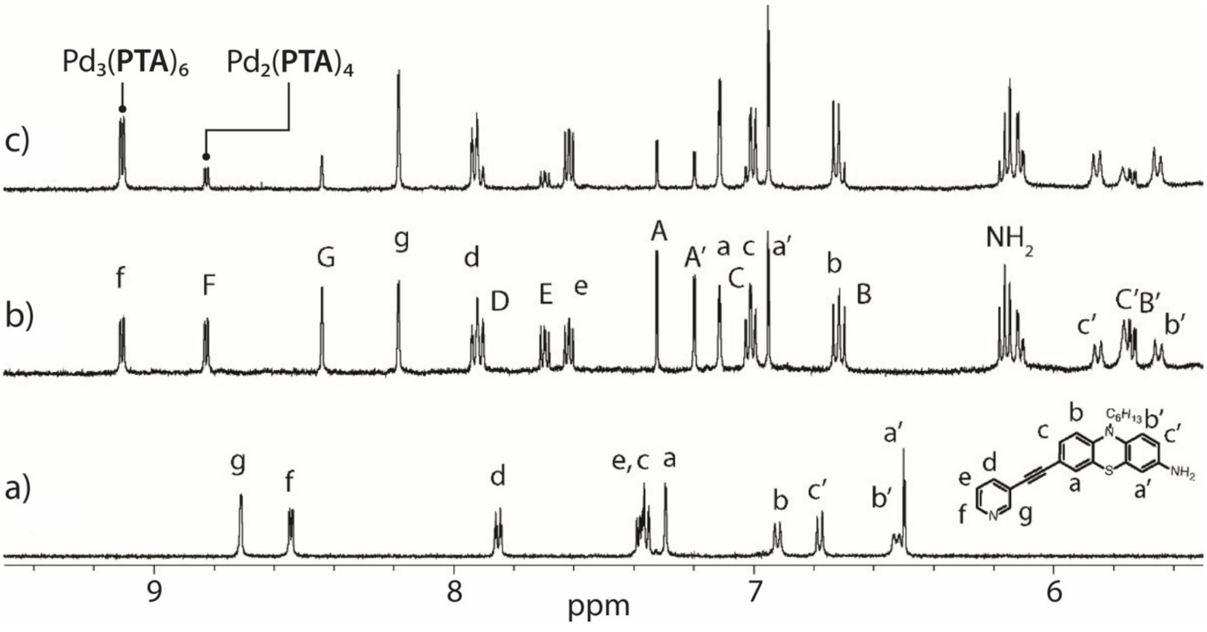

Figure S21. ^{1}H NMR (500 MHz, CD_3CN) spectra of (a) **PTA**, mixture of **PTA** and $[Pd(CH_3CN)_4(BF_4)_2]$ after (b) 30 min and (c) 24 h.

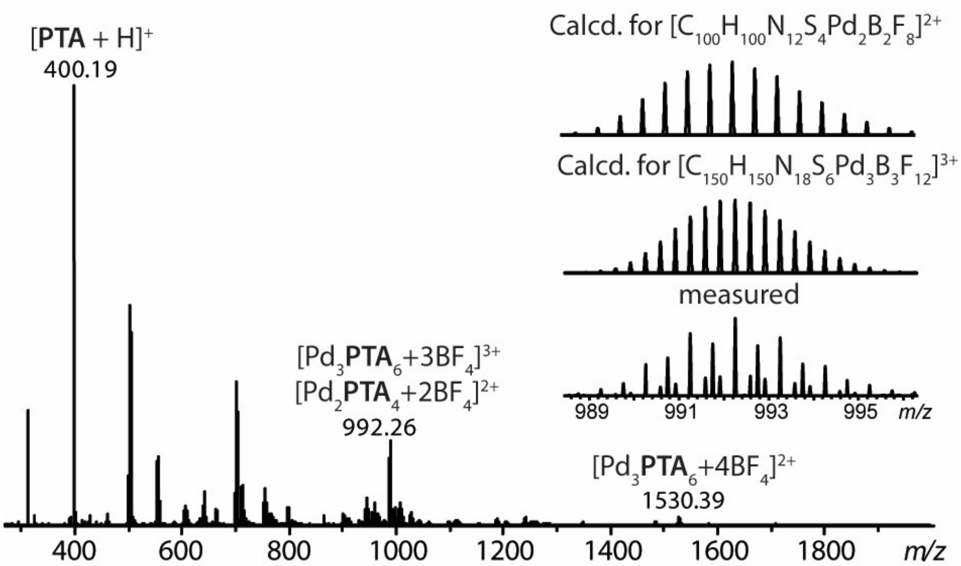

Figure S22. ESI-MS spectrum of the $Pd_n(\mathbf{PTA})_{2n}$ assembly mixture.

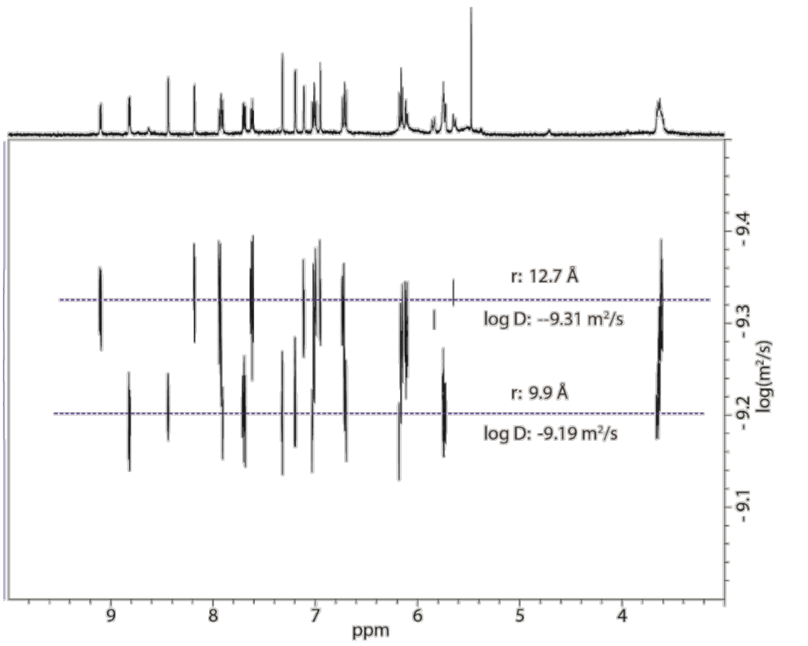

Figure S23: [1]H DOSY (500 MHz, 298K, CD_3CN) spectrum of mixture of Pd_n(**PTA**)$_{2n}$ assemblies. (D(Pd_2(**PTA**)$_4$) = 6.562×10^{-10} m^2s^{-1}, logD(Pd_2(**PTA**)$_4$) = –9.19 m^2s^{-1}, r_H(Pd_2(**PTA**)$_4$) = 9.9 Å; D(Pd_3(**PTA**)$_6$) = 5.148×10^{-10} m^2s^{-1}, logD(Pd_3(**PTA**)$_6$) = –9.31 m^2s^{-1}, r_H(Pd_3(**PTA**)$_6$) = 12.7 Å)

S3.2 Monitoring of $Pt_2Pd_2L_8$ double cage assembly

PTA, [Pt(**PyCHO**)$_4$](BF_4)$_2$, and [Pd(CH_3CN)$_4$](BF_4)$_2$ were combined in CD_3CN at room temperature, and the progress of the complexation reaction was monitored using ^{1}H NMR spectroscopy. Within 10 minutes, the signals corresponding to **PTA** disappeared, and new signals emerged in the 1H NMR spectrum (Figure S24), matching those of the Pd_n(**PTA**)$_{2n}$ assemblies as characterized above (Figure S28). This indicated that rapid complexation of **PTA** with Pd(II) occurred before imine condensation.

Subsequently, signals corresponding to the product Pt_2Pd_2**L**$_8$ began to appear within one hour. Over time, the signals for Pd_n(**PTA**)$_{2n}$ species disappeared as the formation of Pt_2Pd_2**L**$_8$ progressed. It is hypothesized that a kinetically unstable monomeric cage, PtPd**L**$_4$, formed transiently and immediately underwent dimerization to yield the more stable interpenetrated structure. This suggests that the initial assembly between **PTA**

and Pd(II) ions leads to the formation of Pd_n(**PTA**)$_{2n}$, which then disassembles, exposing the amine groups of **PTA** to the aldehyde groups of the Pt(II) precursor. This results in the formation of dynamic covalent imine bonds. Eventually, all components self-assemble to yield the heterometallic Pt_2Pd_2**L**$_8$ complexes, with *oo*-Pt_2Pd_2**L**$_8$ representing the major species.

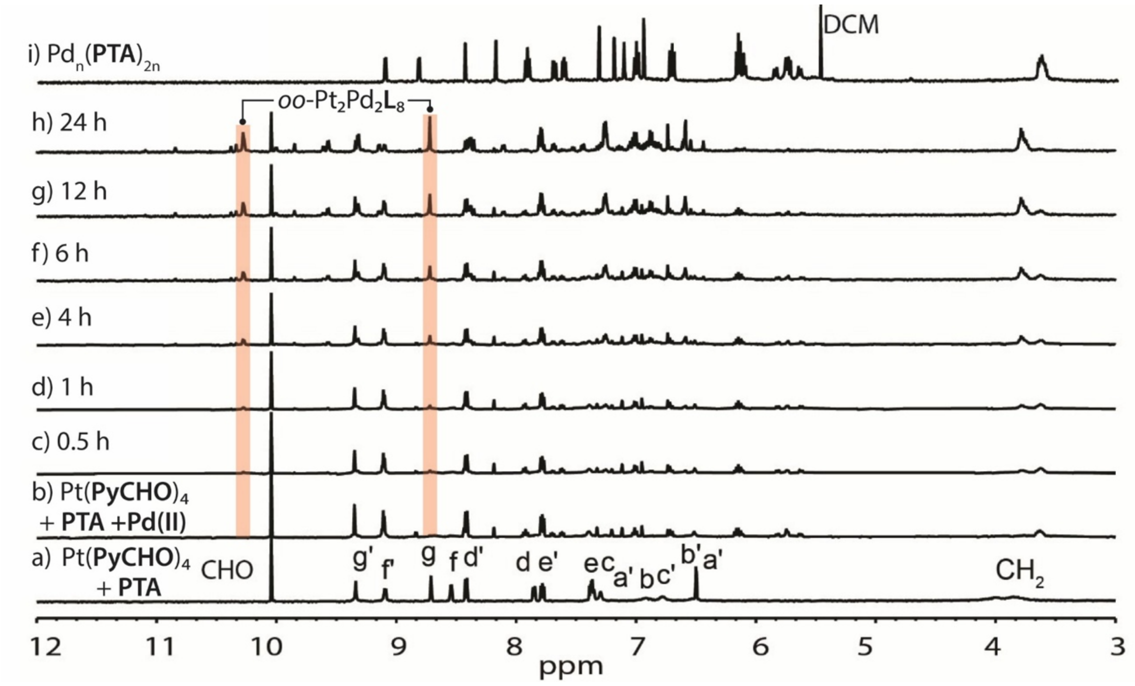

Figure S24. ^{1}H NMR (500 MHz, CD_3CN) spectra of a) mixture of **PTA** and [Pt(**PyCHO**)$_4$]$(BF_4)_2$ (4:1.1 eq.); b) same mixture after addition of 1.0 eq. of $[Pd(CH_3CN)_4(BF_4)_2$ after 10 min, and after c) 30 min, d) 1 h, e) 4 h, f) 6 h, g) 12 h, h) 24 h; i) Mixture of 1 eq. of $[Pd(CH_3CN)_4(BF_4)_2]$ and **PTA** (2 eq.) after 30 min.

S3.3 Possible mechanisms of $Pt_2Pd_2\mathbf{L}_8$ double cages formation

Assuming that the formation and interlocking of $Pt_2Pd_2\mathbf{L}_8$ cages start from the monomeric $PtPd\mathbf{L}_4$ cage, at least three possible mechanisms for the formation of heterometallic interlocked double cages can be proposed (**Scheme S10**).

The first pathway involves encapsulation of cage metal-containing subcomponents within the $PtPd\mathbf{L}_4$ interior, templated by counterions, followed by further self-assembly and interlocking through imine condensation (Scheme S10a). However, for complete conversion of the components to $Pt_2Pd_2\mathbf{L}_8$, the self-assembly within the cage must proceed faster than the formation of the initial $PtPd\mathbf{L}_4$, or the already formed $PtPd\mathbf{L}_4$ must be able to disassemble back into the starting components.

The second pathway involves the formation of $Pt\mathbf{L}_4$ species through the decoordination of a Pd(II) ion from the $PtPd\mathbf{L}_4$ cage (**Scheme S10b**). The resulting $Pt\mathbf{L}_4$ may dimerize and interlock (via re-coordination of Pd(II)) either with itself to yield the *ii*-$Pt_2Pd_2\mathbf{L}_8$ isomer or with a $PtPd\mathbf{L}_4$ cage to yield the *oo*- and *io*-isomers. However, because this pathway requires complete decoordination of a Pd(II) ion from the $PtPd\mathbf{L}_4$ cage, it is therefore considered less probable.

The most probable mechanism is stepwise interlocking through a sequence of ligand de-/recoordination events, resulting in a series of $Pt_2Pd_2\mathbf{L}_8$ intermediates with different degrees of interlocking (**Scheme S10c**). Previously, Hiraoka and co-workers proposed a similar mechanism for the formation of Pd_4L_8 cages.[54, main text] Additionally, in our previous work we showed that the dimerization of a Pd_2L_4 cage may result not only in quadruply interlocked Pd_4L_8 double cages (the same interlocking degree as in this work) but also in less intertwined triply interlocked Pd_4L_8 double cages and cages with different degrees of interlocking were shown to interconvert into one another.[46, main text] We thus presume that the dimerization of the $PdPt\mathbf{L}_4$ monomeric cage precursors mainly proceeds stepwise by de-/recoordination involving the two metal ions that penetrate into the cage interior and eventually become buried within the cage. In the case of the oo-$Pt_2Pd_2\mathbf{L}_8$ isomer, both internal metal ions are labile Pd(II) centers, which

can easily exchange ligands and as such bring the structure toward the quadruply interlocked final product. In contrast, for the io-isomer, only one of the two internal metal ions is labile Pd(II) while the other is the inert Pt(II) center (and for the ii isomer even both inner metals are Pt). Following the discussed mechanism, the probability of ligand exchange processes upon interlocking should be strongly reduced for the io- and ii-isomers, suggesting that their formation should be kinetically hindered. The formation of *ii*-isomer according to the stepwise mechanism appears to be the least probable because it involves the cleavage of only Pt-N bonds.

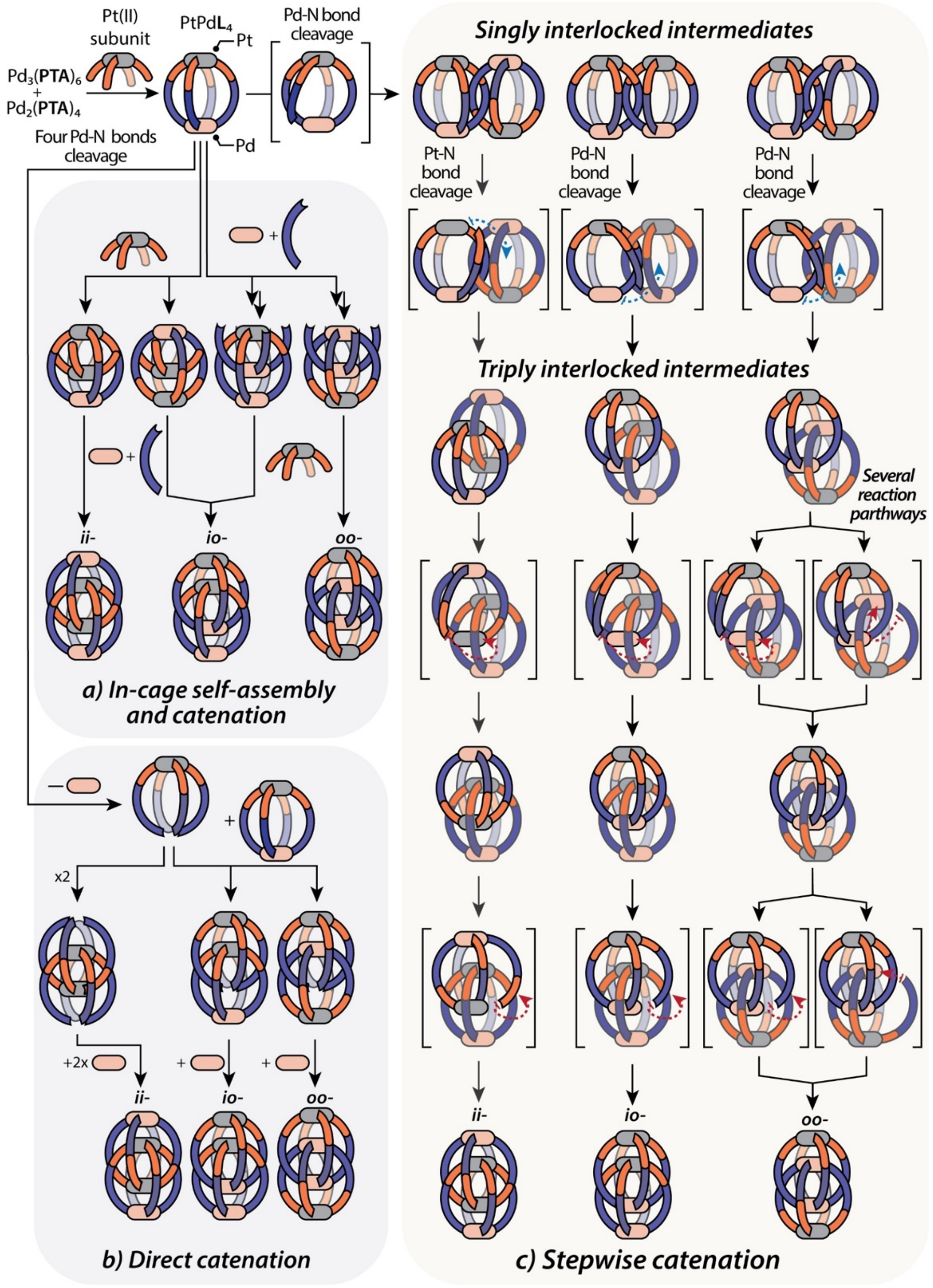

Scheme S10. Possible mechanisms of $Pt_2Pd_2\mathbf{L}_8$ cages formations.

S4 Halide uptake and cage stability study

The halide uptake by $Pt_2Pd_2\mathbf{L}_8$ were studied by titrating a solution of the halide anions (*n*-Bu_4N^+ salts – TBACl and TBABr – in CD_3CN, 20 mM) step by step into 500µL of a 0.35mM solution of the cages in CD_3CN in an NMR tube.

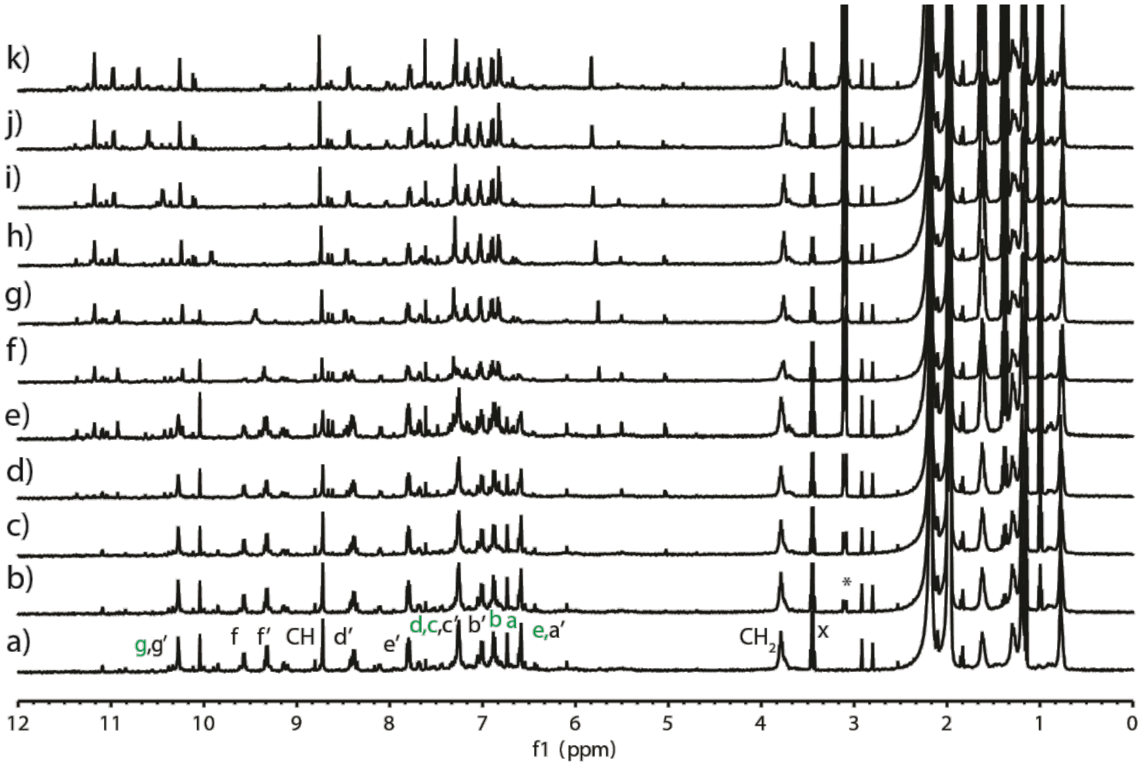

Figure S25. ^{1}H NMR (500 MHz, CD_3CN) spectra of a) $[Pt_2Pd_2\mathbf{L}_8](BF_4)_8$, after addition of b) 0.25 eq., c) 0.5 eq., d) 1.0 eq., e) 1.5 eq., f) 2 eq., g) 3 eq., h) 6 eq., i) 9 eq., j) 12 eq., k) 24 eq. of TBACl (10 mM solution in CD_3CN) with respect to $[Pt_2Pd_2\mathbf{L}_8](BF_4)_8$. ("*" is a CH_2 signal of TBACl, and "x" is diethyl ether residual solvent signal).

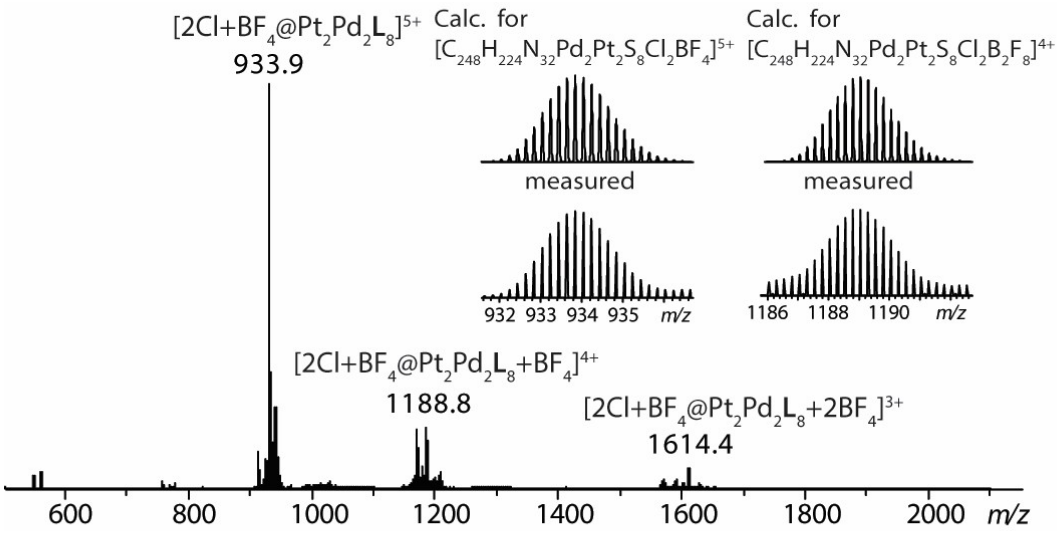

Figure S26. ESI-MS spectrum of $Pt_2Pd_2\mathbf{L}_8$ after addition of 3 eq. of TBACl.

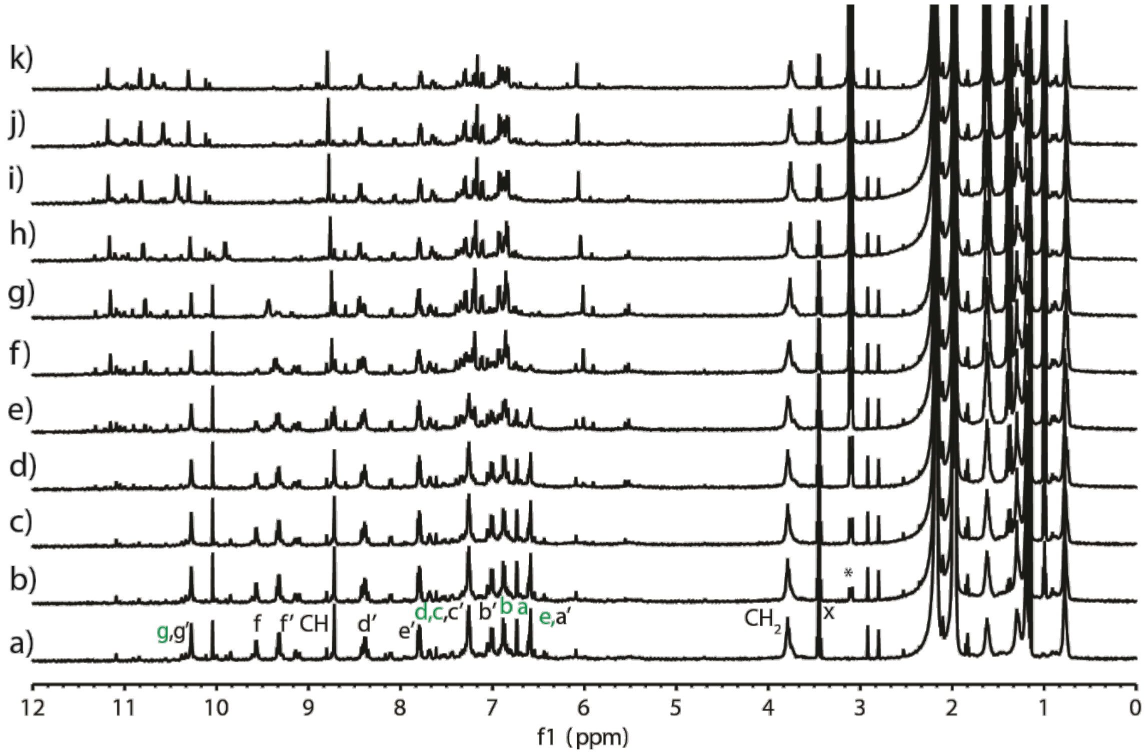

Figure S27. ^{1}H NMR (500 MHz, CD_3CN) spectra of a) $[Pt_2Pd_2\mathbf{L}_8](BF_4)_8$, after addition of b) 0.25 eq., c) 0.5 eq., d) 1.0 eq., e) 1.5 eq., f) 2 eq., g) 3 eq., h) 6 eq., i) 9 eq., j) 12 eq., k) 24 eq. of TBABr (10 mM solution in CD_3CN) with respect to $[Pt_2Pd_2\mathbf{L}_8](BF_4)_8$. ("*" is a CH_2 signal of TBABr, and "x" is diethyl ether residual solvent signal).

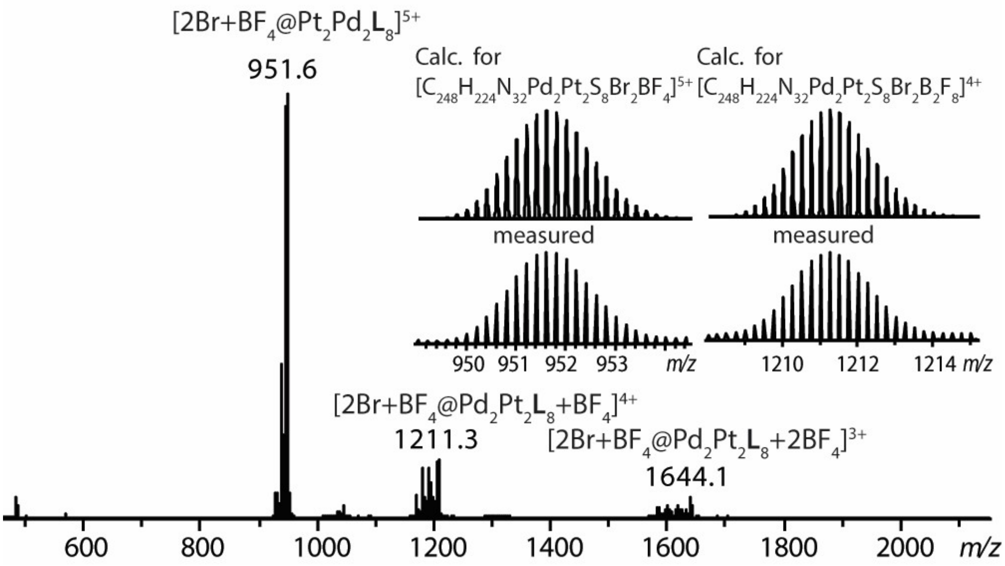

Figure S28. ESI-MS spectrum of Pt_2Pd_2**L**$_8$ after addition of 3 eq. of TBABr.

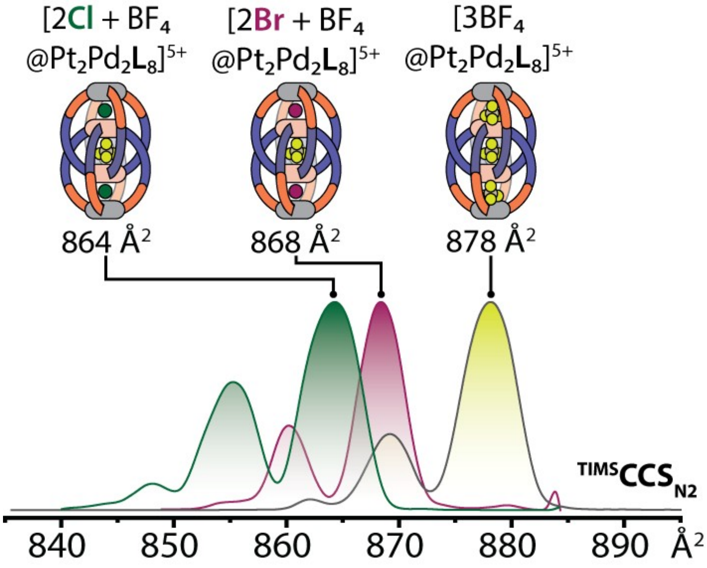

Figure S29. Comparison of the TIMS mobilograms for $[3BF_4@Pt_2Pd_2$**L**$_8]^{5+}$ (yellow), $[2Cl+BF_4@Pt_2Pd_2$**L**$_8]^{5+}$ (green), $[2Br+BF_4@Pt_2Pd_2$**L**$_8]^{5+}$ (red) ions. Estimated TIMS isomer ratios (*oo*:*io*:*ii*): $[3BF_4@Pt_2Pd_2$**L**$_8]^{5+}$ – 75:22:3, $[2Cl+BF_4@Pt_2Pd_2$**L**$_8]^{5+}$ – 60:35:5, $[2Br+BF_4@Pt_2Pd_2$**L**$_8]^{5+}$ – 74:24:2. Experimentally determined collisional cross section (CCS) values are annotated to the major double cage isomer, each. The experiments reflect well the expected trend in double cage dimensions as a function of the sizes of the anions in the outer pockets.[8,10,main text]

S5 Halide abstraction studies

Halide abstraction reactions from haloorganics by the $Pt_2Pd_2\mathbf{L}_8$ cage were studied by adding 2 eq. of triphenylsilyl chloride (Ph_3SiCl), benzhydryl chloride (Ph_2CHCl), or benzhydryl bromide (Ph_2CHBr) (in CD_3CN, 20 mM) to 500 μL of a 0.35 mM CD_3CN solution of $Pt_2Pd_2\mathbf{L}_8$ in an NMR tube.

For the samples containing triphenylsilyl chloride (**Figure S30e**) and benzhydryl bromide (**Figure S30i**), ^{1}H NMR spectra were recorded after 1 hour at room temperature. For the samples containing benzhydryl chloride, ^{1}H NMR spectra were recorded after 18 hours at room temperature, and then again after heating for 2 hours at 70 °C (**Figures S30f** and **S30g**).

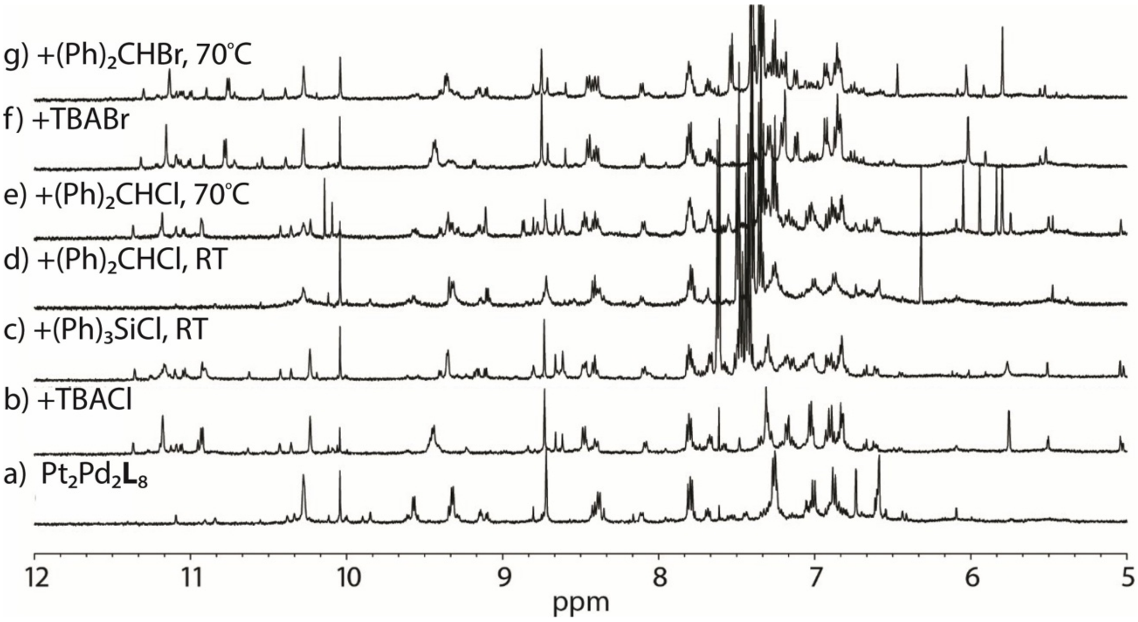

Figure S30. ^{1}H NMR (500 MHz, CD_3CN) spectra of a) $Pt_2Pd_2\mathbf{L}_8$, after addition of: b) 2 eq. of TBACl (at room temperature), c) 2 eq. of triphenylsilyl chloride (at room temperature) d) benzhydryl chloride (at room temperature, 18 h), e) benzhydryl chloride (at 70 ˚C, 2 h), f) TBABr (at room temperature), and g) benzhydryl bromide (at room temperature, 1 h)

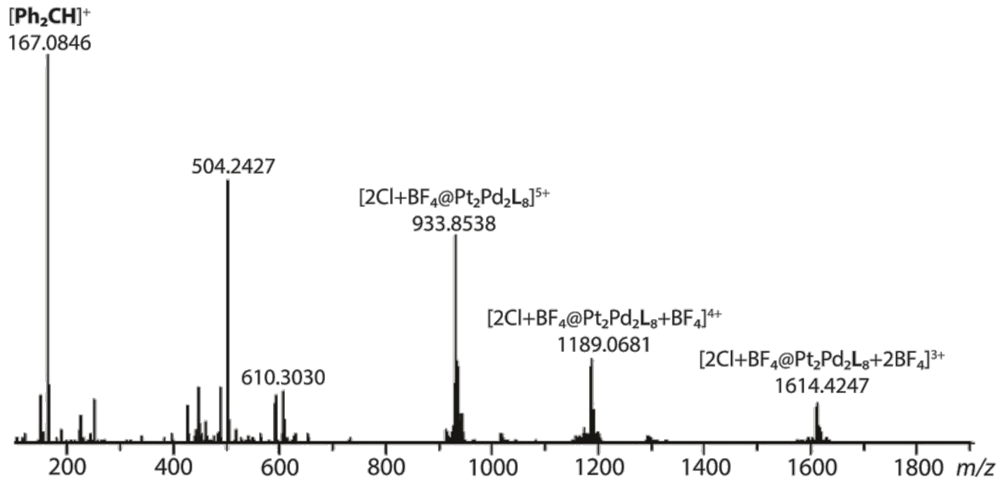

Figure S31. ESI-MS spectrum of $[Pt_2Pd_2\mathbf{L}_8](BF_4)_8$, after addition of 2 eq. of benzhydryl chloride at 70 ˚C, 2 h. Showing the signals for $[2Cl+BF_4@[Pt_2Pd_2\mathbf{L}_8]^{5+}$ and benzhydryl cation signals.

S6 X-ray crystallography

Orange block crystals of Pt_2Pd_2**L**$_8$ were grown by slow vapor diffusion of benzene into a 0.3 mM solution the $[Pt_2Pd_2$**L**$_8](BF_4)_8$ assembly and 1eq. of $(n\text{-}Bu)_4Cl$ in CD_3CN. The crystals were stored at cryogenic temperatures in dry shippers, which were used to safely transport them to the macromolecular beamline P11 at PETRA III, DESY, Hamburg, Germany.

A wavelength of λ = 1.033 Å was selected using a liquid nitrogen-cooled double crystal monochromator. Single-crystal X-ray diffraction data were collected at 100(2) K on a single-axis goniometer equipped with an Oxford Cryostream 800 and an Eiger 2X 16M detector. A total of 3600 diffraction images were collected during a 360° φ sweep at a detector distance of 154 mm, with 100% filter transmission, a 0.1° step width, and an exposure time of 0.5 seconds per image.

Data integration and reduction were undertaken using XDS.[64] The structure was solved by direct methods using SHELXT[65] and refined by full-matrix least-squares methods against F^2 by SHELXL[66] in the OLEX2 program package.[67, 68] All non-hydrogen atoms were refined with anisotropic displacement parameters. The hydrogen atoms were refined isotropically on calculated positions using a riding model with their U_{iso} values constrained to 1.2 times. Crystal data and structure refinement for Pt_2Pd_2**L**$_8$ are given in **Table S1**.

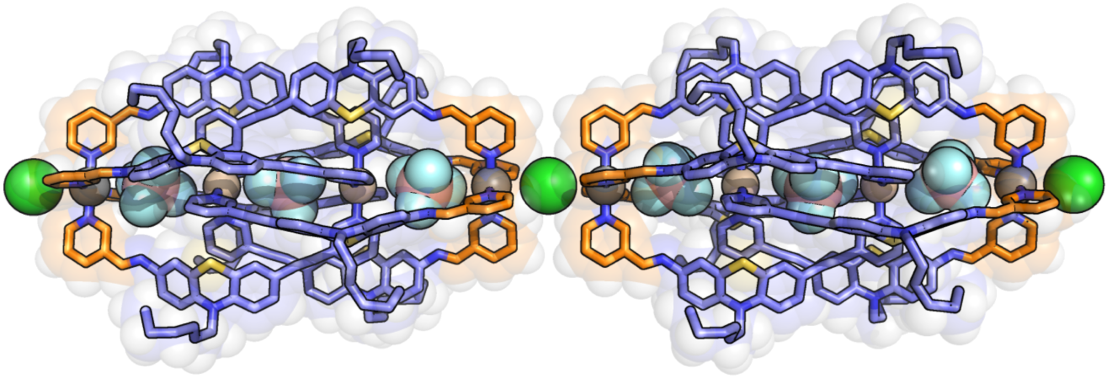

Figure S32. Crystal packing mode of *oo*-$Pt_2Pd_2\mathbf{L}_8$ molecules involving Cl^- counter-ions.

Table S1. Crystal data and structure refinement for $Pt_2Pd_2\mathbf{L}_8$.

Structure	$Pt_2Pd_2\mathbf{L}_8$
Identification code	sg88b_12KeV
CCDC code	2453736
Empirical formula	$C_{320}H_{296}B_7ClF_{28}N_{32}Pd_2Pt_2S_8$
Formula weight	6092.45
Temperature/K	100
Crystal system	tetragonal
Space group	P4/nnc
a/Å	21.2120(18)
b/Å	21.2120(18)
c/Å	32.253(5)
α/°	90
β/°	90
γ/°	90
Volume/Å^3	14512(3)
Z	2
ρ_{calc}g/cm^3	1.394
μ/mm^{-1}	2.380
F(000)	6240.0
Crystal size/mm^3	0.02 × 0.02 × 0.01
Radiation	synchrotron (λ = 1.033)
2Θ range for data collection/°	3.34 to 53.368

Index ranges	-15 ≤ h ≤ 15, -16 ≤ k ≤ 16, -26 ≤ l ≤ 26
Reflections collected	48361
Independent reflections	2305 [R_{int} = 0.0627, R_{sigma} = 0.0236]
Data/restraints/parameters	2305/890/655
Goodness-of-fit on F^2	1.583
Final R indexes [I>=2σ (I)]	R_1 = 0.1272, wR_2 = 0.3175
Final R indexes [all data]	R_1 = 0.1420, wR_2 = 0.3391
Largest diff. peak/hole / e Å^{-3}	2.40/-0.52

S7 Theoretical calculations

The geometry optimization and single-point energy calculations of the studied cages were conducted using the ORCA 6.0 software.[69] The initial structures for the calculations were built based on the obtained X-ray structure of $Pt_2Pd_2\mathbf{L}_8$. Three isomers of $Pt_2Pd_2\mathbf{L}_8$ (with three BF_4^- anions placed in the cage cavities) were optimized on r^{2}scan-3c[47,main text] level in the gas phase. The resulting gas-phase structures of the $Pt_2Pd_2\mathbf{L}_8$ isomers were used to calculate theoretical collision cross section (CCS) values using the Collidoscope software, version 1.4.[70] To evaluate the relative energies of the $Pt_2Pd_2\mathbf{L}_8$ isomers, the structures were further optimized at the r^{2}SCAN-3c level using the SMD(CH_3CN) solvation model.[48,main text] Same level of calculation were used for optimization of $PtPd\mathbf{L}_4$, Pd_2(**PTA**)$_4$, Pd_3(**PTA**)$_6$, and 2X+3BF_4@$Pt_2Pd_2\mathbf{L}_8$ (X = Cl or Br) cages. The Cartesian atomic coordinates for the optimized model structures are provided as supplementary XYZ files.

Final single-point energy calculations were carried out at the ωB97X-D4/def2-TZVPP/SMD-DRACO(CH_3CN) level,[49–52,main text] while thermostatistical corrections (G_{TRV}) using the modified rigid rotor harmonic oscillator (mRRHO) approach[53,main text] were obtained from harmonic frequency calculations at the GFN2-xTB[71]/ALPB[72](CH_3CN) level level using the xTB software, version 6.7.1[73] (**Table S2**).

The optimized structures of the $Pt_2Pd_2\mathbf{L}_8$ isomers were then split without further optimization to analyze the contributions of different fragments to the stabilization of the interlocked structures (**Figure S33**). The first fragmentation pattern was generated by removing the ligand backbones, leaving only the $[M(Py)_4]^{2+}$ and BF_4^- units (hydrogens were added in place of the substituents on the pyridine ligands). The second pattern was obtained by omitting all Pt(II), Pd(II), and BF_4^- ions from the structures, leaving only the organic ligands. The energies of the fragments were calculated at the same level of theory as the single-point energy calculations for the full cage structures (**Table S2**). The resulting energy differences between the isomers,

as well as between their respective fragments, are summarized in **Table S3** and **Figure S33**.

Based on the calculations results (**Table S3** and **Figure S33**), the *oo*-Pt_2Pd_2**L**$_8$ isomer is approximately 20 kJ/mol more stable than the symmetric *ii*-isomer and only about 3 kJ/mol less stable than the asymmetric *io*-isomer. Notably, a comparison based solely on electronic energies (E_{SCF}) suggests that the *oo*-isomer is more stable than both the *io*- and *ii*-isomers (ΔE_{SCF} = 9 and 26 kJ/mol, respectively), whereas the inclusion of dispersion corrections slightly favors the *io*-isomer over the *oo*-isomer. The application of thermostatistical corrections (G_{TRV}) has little effect on the energy difference between the *oo*- and *io*-isomers but leads to greater destabilization (5 kJ/mol) of the *ii*-isomer compared to the *oo*-one.

Fragmentation analysis and comparison of fragment energies across the isomers revealed that the linear alternating arrangement of four $[M(Py)_4]^{2+}$ units and three BF_4^- ions (excluding the ligand backbones; see Figure S33), is 8 and 22 kJ/mol more favorable in the *oo*-isomer than in the io- and ii-isomers, respectively. The more detailed analysis has shown that the differences in the stability of various ion arrangements are governed by both ion–ion interactions and the strain within the $[M(Py)_4]^{2+}$ units induced by ligand arrangement. The Pt-Pd-Pd-Pt arrangement in *oo*-isomer exhibits both stronger interactions of $[M(Py)_4]^{2+}$ units and BF_4^- anions as well as lesser strain within the $M(Py)_4$ units (Table S4, Figure S34). Additionally, the comparison of the $[M(Py)_4]^{2+}$–BF_4^-–$[M(Py)_4]^{2+}$ fragments extracted from the cage structures (Figure S34) has revealed that binding BF_4^- anion between $[M(Py)_4]^{2+}$ units is increasing in the row: $[Pt(Py)_4]^{2+}$–BF_4^-–$[Pt(Py)_4]^{2+}$ < $[Pt(Py)_4]^{2+}$–BF_4^-–$[Pd(Py)_4]^{2+}$ < $[Pd(Py)_4]^{2+}$–BF_4^-–$[Pd(Py)_4]^{2+}$. Because the *oo*-isomer contains two $[Pt(Py)_4]^{2+}$–BF_4^-–$[Pd(Py)_4]^{2+}$ and one $[Pd(Py)_4]^{2+}$–BF_4^-–$[Pd(Py)_4]^{2+}$ fragments, it binds BF_4^- more strongly than the other isomers. This likely makes a major contribution to the stabilization of the *oo*-isomer. Conversely, the ligand arrangement (excluding the metal and BF_4^- ions) is more favorable in the *io*- and *ii*-isomers by 20 and 8 kJ/mol, respectively, compared

to the *oo*-isomer, likely due to variations in ligand–ligand noncovalent interaction patterns and ligand strain.

The analysis of isomeric Pt_2Pd_2**L_8** cages containing two halide anions in the outer pocket (2X+3BF_4@Pt_2Pd_2**L_8** (X = Cl or Br)) revealed that for both chloride and bromide anions the *oo*-isomer is on 31–41 kJ/mol is more stable than corresponding io- and ii-isomers (**Table S5**).

TableS2. Calculated energies (Hartree) for $Pt_2Pd_2\mathbf{L}_8$ isomers and their fragments.

Energy	Full structure	Ion arrangement	Ligand arrangement
oo-$Pt_2Pd_2\mathbf{L}_8$			
E_{SCF}	-14719.172967	-5741.124789	-12950.928514
$E_{disp.corr.}$	-0.215109	-0.063840	-0.186512
E_{total}	-14719.388077	-5741.188629	-12951.115026
G_{TRV}	2.704339	-	-
G^{298}	-14716.683738	-	-
io-$Pt_2Pd_2\mathbf{L}_8$			
E_{SCF}	-14719.169667	-5741.122522	-12950.931523
$E_{disp.corr.}$	-0.219527	-0.063205	-0.190989
E_{total}	-14719.389194	-5741.185726	-12951.122512
G_{TRV}	2.704269	-	-
G^{298}	-14716.684925	-	-
ii-$Pt_2Pd_2\mathbf{L}_8$			
E_{SCF}	-14719.162742	-5741.116522	-12950.927135
$E_{disp.corr.}$	-0.219507	-0.063575	-0.191058
E_{total}	-14719.382249	-5741.180096	-12951.118194
G_{TRV}	2.706176	-	-
G^{298}	-14716.676073	-	-
$E_{disp.corr.}$: Dispersion correction energy; G_{TRV}: thermostatistical correction<br>$E_{total} = E_{SCF} + E_{disp.corr.}$; $G^{298} = E_{total} + G_{TRV}$			

TableS3. Calculated energy differences (kJ/mol) between $Pt_2Pd_2\mathbf{L}_8$ isomers and their fragments.

Energy	Full structure	Ion arrangement	Ligand arrangement
$\Delta E(oo - io)$			
ΔE_{SCF}	-8.7	-6.0	7.9
$\Delta E_{disp.corr.}$	11.6	-1.7	11.8
ΔE_{total}	2.9	-7.6	19.7
ΔG_{TRV}	0.2	-	-
$\boldsymbol{\Delta G^{298}}$	3.1	-	-
$\Delta E(oo - ii)$			
ΔE_{SCF}	-26.8	-21.7	-3.6
$\Delta E_{disp.corr.}$	11.5	-0.7	11.9
ΔE_{total}	-15.3	-22.4	8.3
ΔG_{TRV}	-4.8	-	-
$\boldsymbol{\Delta G^{298}}$	-20.1	-	-

TableS4. Calculated electronic (Hartree), association (E_a), strain (E_{strain}) energies (kJ/mol) and their differences (kJ/mol) for the different ion arrangement in Pt_2Pd_2**L**$_8$ isomers.

Structure	*oo-*	*io-*	*ii-*
Association of $[M(Py)_4]_4(BF_4)_3]^{3+}$ ($E_a = E_{full} - \Sigma E_{fragment}$)			
E_{full}	-5741.124789	-5741.122522	-5741.116522
$\Sigma E_{fragment}$	-5741.09290	-5741.09140	-5741.08937
E_a	-251.3	-247.6	-238.2
Strain in $M(Py)_4$ units ($E_{strain} = E_{cage\ geom.} - E_{opt\ geom.}$)			
$\Sigma E_{cage\ geom.}$	-4466.992973	-4466.991476	-4466.989444
$\Sigma E_{opt\ geom.}$	-4467.007881	-4467.007881	-4467.007881
ΣE_{strain}	39.1	43.1	48.4
Differences in association, strain, and electronic energies			
ΔE_a *(oo – xx)*	-	-3.7	-13.1
ΔE_{strain} *(oo – xx)*	-	-3.9	-9.3
ΔE_{full} *(oo – xx)*	-	-7.6	-22.4

TableS5. Calculated energies (Harteree) and their differences (kJ/mol) between Pt_2Pd_2**L**$_8$ isomers with different anions in the outer pockets.

Energy	*oo*	*io*	*ii*	Δ*E(oo – io)*	Δ*E(oo – ii)*
$3BF_4@[Pt_2Pd_2$**L**$_8$					
ΔE_{SCF}	-14719.172967	-14719.169667	-14719.162742	-8.7	-26.8
$\Delta E_{disp.corr.}$	-0.215109	-0.219527	-0.219507	11.6	11.5
ΔE_{total}	-14719.388077	-14719.389194	-14719.382249	2.9	-15.3
ΔG_{TRV}	2.704339	2.704269	2.706176	0.2	-4.8
**ΔG^{298}**	-14716.683738	-14716.684925	-14716.676073	3.1	-20.1
2**Cl**$+BF_4@[Pt_2Pd_2$**L**$_8$					
ΔE_{SCF}	-14790.526592	-14790.512723	-14790.512427	-36.4	-37.2
$\Delta E_{disp.corr.}$	-0.214419	-0.218059	-0.217768	9.6	8.8
ΔE_{total}	-14790.741011	-14790.730783	-14790.730195	-26.9	-28.4
ΔG_{TRV}	2.683744	2.685473	2.684877	-4.5	-3.0

**Δ*G*^{298}**	-14788.057267	-14788.045310	-14788.045318	-31.4	-31.4
2**Br**+BF_4@[Pt_2Pd_2**L**$_8$					
ΔE_{SCF}	-19018.507184	-19018.489882	-19018.488652	-45.4	-48.7
Δ$E_{disp.corr.}$	-0.213831	-0.217875	-0.216924	10.6	8.1
ΔE_{total}	-19018.721015	-19018.707757	-19018.705576	-34.8	-40.5
ΔG_{TRV}	2.683198	2.683133	2.683284	0.2	-0.2
**Δ*G*^{298}**	-19016.037818	-19016.024624	-19016.022291	-34.6	-40.8

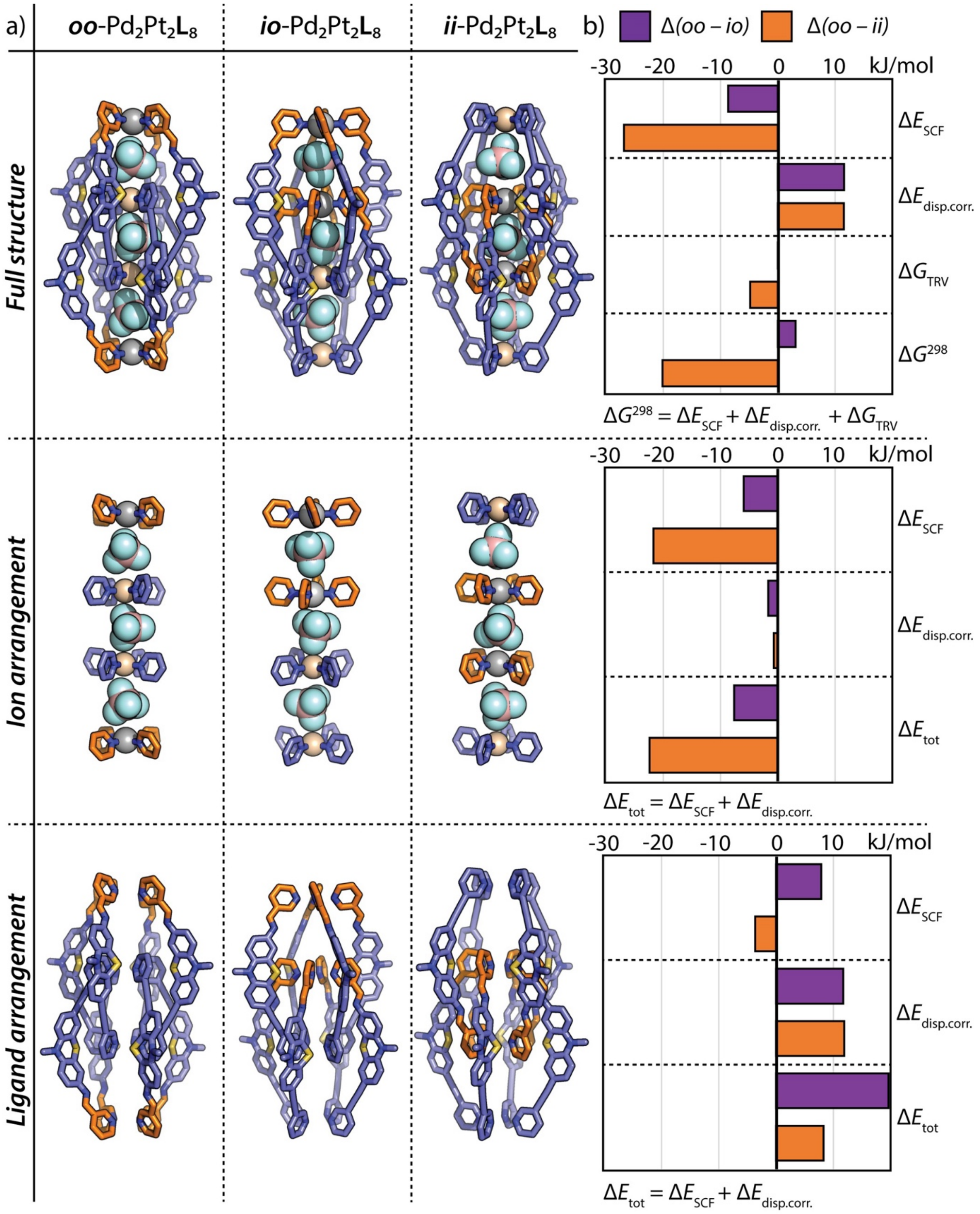

Figure S33. (a) Fragmentation scheme of $Pt_2Pd_2\mathbf{L}_8$ isomers for the energy calculations and (b) bar diagram of energy differences between $Pt_2Pd_2\mathbf{L}_8$ isomers for the different terms and fragments. Data is taken from Table S3.

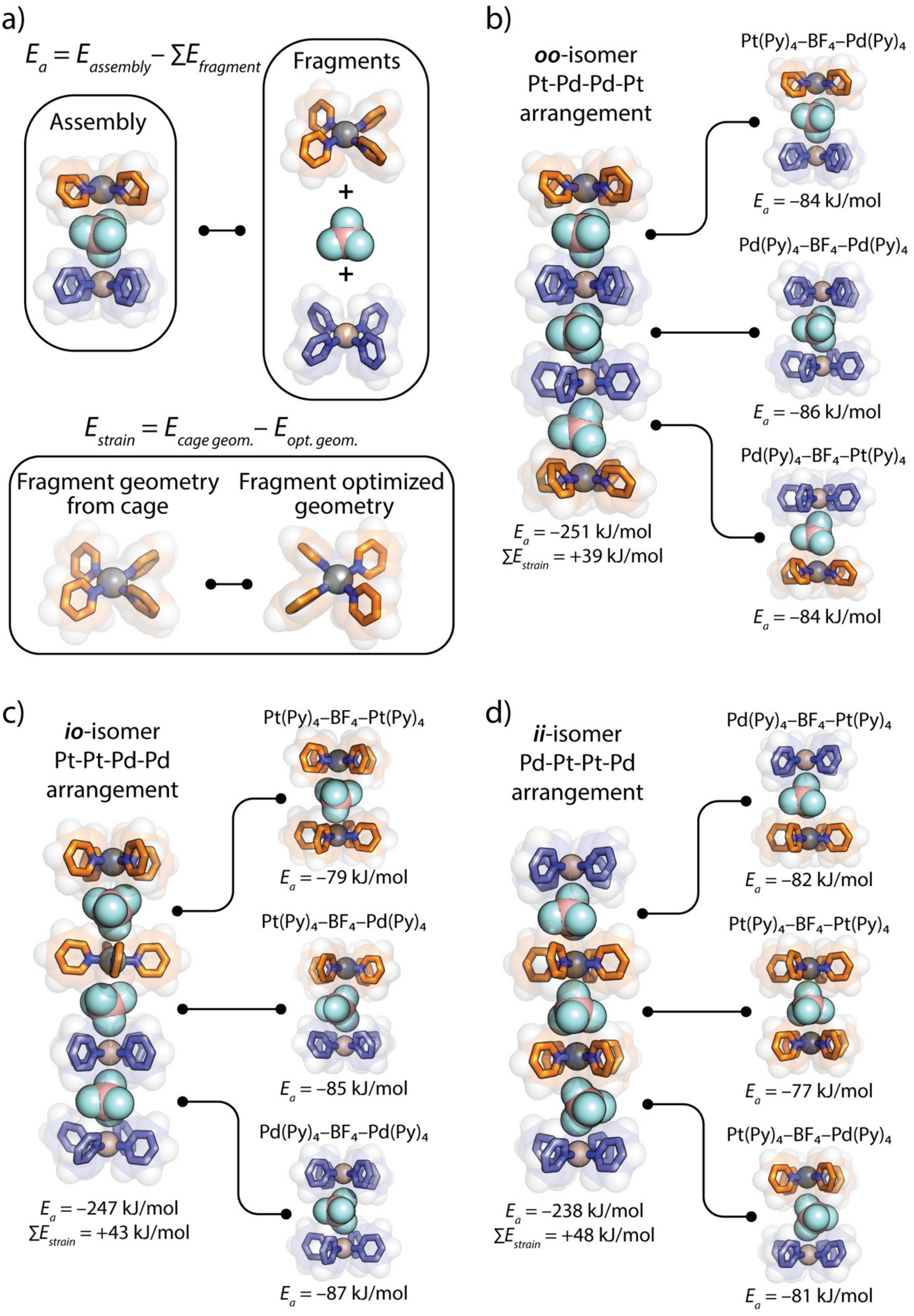

Figure S34. (a) Estimation of association and strain energies in ion arrangements of *oo*-(b), *io*-(c), *ii*-Pt_2Pd_2**L**$_8$ (d) isomers. Data is taken from Table S4.

S8 References

[60] E. O. Stejskal and J. E. Tanner, "Spin Diffusion Measurements: Spin Echoes in the Presence of a Time-Dependent Field Gradient" *J. Chem. Phys.* **1965**, *42*, 288-292.

[61] J. E. Tanner and E. O. Stejskal, "Restricted Self-Diffusion of Protons in Colloidal Systems by the Pulsed-Gradient, Spin-Echo Method" *J. Chem. Phys.* **1968**, *49*, 1768-1777.

[62] E. A. Mason and H. W. Schamp, "Mobility of gaseous Ions in weak electric fields" *Ann. Phys.* **1958**, *4*, 233-270.

[63] M. Sailer, A. W. Franz and T. J. J. Müller, "Synthesis and Electronic Properties of Monodisperse Oligophenothiazines" *Chem. Eur. J.* **2008**, *14*, 2602-2614.

[64] W. Kabsch, "Xds" *Acta Crystallogr., Sect. D: Biol. Crystallogr.* **2010**, *66*, 125-132.

[65] G. Sheldrick, "SHELXT - Integrated space-group and crystal-structure determination" *Acta Crystallographica Section A* **2015**, *71*, 3-8.

[66] G. M. Sheldrick, "A short history of SHELX" *Acta Crystallogr., Sect. A: Found. Crystallogr.* **2008**, *64*, 112-122.

[67] L. J. Bourhis, O. V. Dolomanov, R. J. Gildea, J. A. K. Howard and H. Puschmann, "The anatomy of a comprehensive constrained, restrained refinement program for the modern computing environment - Olex2 dissected" *Acta Crystallogr., Sect. A: Found. Crystallogr.* **2015**, *71*, 59-75.

[68] O. Dolomanov, L. Bourhis, R. Gildea, J. Howard and H. Puschmann, "OLEX2: a complete structure solution, refinement and analysis program" *J. Appl. Crystallogr.* **2009**, *42*, 339-341.

[69] F. Neese, "Software update: The ORCA program system—Version 5.0" *Wiley Interdiscip. Rev.:Comput. Mol. Sci.* **2022**, *12*, e1606.

[70] S. A. Ewing, M. T. Donor, J. W. Wilson and J. S. Prell, "Collidoscope: An Improved Tool for Computing Collisional Cross-Sections with the Trajectory Method" *J. Am. Soc. Mass Spectrom.* **2017**, *28*, 587-596.

[71] C. Bannwarth, S. Ehlert and S. Grimme, "GFN2-xTB—An Accurate and Broadly Parametrized Self-Consistent Tight-Binding Quantum Chemical Method with

Multipole Electrostatics and Density-Dependent Dispersion Contributions" *J. Chem. Theory Comput.* **2019**, *15*, 1652-1671.

[72] S. Ehlert, M. Stahn, S. Spicher and S. Grimme, "Robust and Efficient Implicit Solvation Model for Fast Semiempirical Methods" *Journal of Chemical Theory and Computation* **2021**, *17*, 4250-4261.

[73] C. Bannwarth, E. Caldeweyher, S. Ehlert, A. Hansen, P. Pracht, J. Seibert, S. Spicher and S. Grimme, "Extended tight-binding quantum chemistry methods" *Wiley Interdiscip. Rev.:Comput. Mol. Sci.* **2021**, *11*, e1493.
